# Allelic variation in an expansin, *MdEXP-A1*, contributes to flesh firmness at harvest in apples

**DOI:** 10.1186/s43897-024-00121-3

**Published:** 2025-01-20

**Authors:** Qiufang Su, Yifeng Feng, Xianglu Li, Zidun Wang, Yuanwen Zhong, Zhengyang Zhao, Huijuan Yang

**Affiliations:** 1 State Key Laboratory of Crop Stress Biology for Arid Areas, College of Horticulture, Northwest A&F University, Yangling, 712100 Shaanxi China; 2 College of Horticulture and Forestry, Tarim University, Alaer, 843300 Xinjiang China; 3 Liaoning Institute of Pomology, Yingkou, 115009 China

**Keywords:** Expansin, Cell wall loosening, Apple, Firmness at harvest, Transcriptional regulation

## Abstract

Flesh firmness is a core quality trait in apple breeding because of its correlation with ripening and storage. Quantitative trait loci (QTLs) were analyzed through bulked segregant analysis sequence (BSA-seq) and comparative transcriptome analysis (RNA-seq) to explore the genetic basis of firmness formation. In this study, phenotypic data were collected at harvest from 251 F_1_ hybrids derived from ‘Ruiyang’ and ‘Scilate’, the phenotype values of flesh firmness at harvest were extensively segregated for two consecutive years. A total of 11 candidate intervals were identified on chromosomes 03, 05, 06, 07, 13, and 16 via BSA-seq analysis. We characterized a major QTL on chromosome 16 and selected a candidate gene encoding expansin *MdEXP-A1* by combining RNA-seq analysis. Furthermore, the genotype of Del-1166 (homozygous deletion) in the *MdEXP-A1* promoter was closely associated with the super-hard phenotype of F_1_ hybrids, which could be used as a functional marker for marker-assisted selection (MAS) in apple. Functional identification revealed that *MdEXP-A1* positively expedited fruit softening in both apple fruits and tomatoes that overexpressed *MdEXP-A1*. Moreover, the promoter sequence of TE-1166 was experimentally validated containing two binding motifs of MdNAC1, and the absence of the *MdEXP-A1* promoter fragment reduced its transcription activity. MdNAC1 also promotes the expression of *MdEXP-A1*, indicating its potential modulatory role in quality breeding. These findings provide novel insight into the genetic control of flesh firmness by *MdEXP-A1*.

**Supplementary Information:**

The online version contains supplementary material available at 10.1186/s43897-024-00121-3.

## Core

This study demonstrated the role of *MdEXP-A1* in the formation of flesh firmness, and the genetic variation of TE-1166 was significantly correlated with flesh firmness at harvest, which could be used as a potential molecular marker for firmness traits at maturity to assist breeding. The deletion of TE-1166 reduced the expression of adjacent gene *MdEXP-A1* and inhibited the decline of flesh firmness. The transcriptional regulation of *MdEXP-A1* by MdNAC1 further affected the flesh firmness traits.

## Gene & accession numbers

The transcriptome date during development were derived from Liu et al. (2021) and are available in the NCBI database: the accession number is PRJNA728501. The sequence data used in this study can be found in the GenBank data libraries under the following accession numbers: *MdEXP-A1* (MD16G1070600), *MdNAC1* (MD13G1069200), *MdACS1* (MD15G1302200), *MdACO1* (MD10G1328100), and *MdPG1* (MD10G1179100).

## Introduction

Fruit ripening and softening are elaborate and highly coordinated programs that accompany dramatic physicochemical changes (Han et al. 2016; Chen et al. 2020). Flesh firmness is an intrinsic quality trait that has an essential influence on consumer preferences and is also an indicator of harvest date and shelf life. A texture that is too soft or overly hard is not desirable (Hu et al. 2020). Excessive softening not only enhances disease susceptibility but also greatly limits transportability and storability (Su et al. 2023). As a quantitative genetic trait, flesh firmness is controlled by multiple genetic effects and external environmental factors (Chagné et al*.* 2014). Despite a few decades of studies that have explored the physiological or genetic mechanisms underlying fruit softening, their application in marker-assisted selection (MAS) remains limited (Wu et al. 2021b). A clear and systematic understanding of the unique genetic mechanisms of ripening and softening is highly important for providing guidance for genetic improvement.

Apples (*Malus* × *domestica* Borkh) are widely appreciated and grown in temperate regions (Duan et al. 2017). Studies on apple segregation population have identified plenty of quantitative trait loci (QTLs) for flesh firmness or softening on chromosomes 01, 03, 06, 08, 10, 15, and 16 (Longhi et al*.* 2012; Kumar et al. 2013; Bink et al. 2014; Di Guardo et al. 2017; Chagné et al*.* 2014). However, in the case of wide candidate intervals, the determination of core variations in key traits remains challenging (Guo et al. 2020). To date, several crucial genetic variations, especially in *MdACS1*, *MdACO1*, *MdPG1*, and *MdEXP7*, have presented an association between flesh softening and genetic control (Costa et al. 2005; Costa et al*.* 2008; Costa et al*.* 2010). However, increasing evidence has confirmed that genetic diversity can explain only limited phenotypic variation (Nybom et al. 2013; Wu et al. 2021a). With the abandance of high-quality genomes, many studies have suggested that structural variations (SVs) largely contribute to phenotypic variance in diverse species (Li et al. 2023a; Wang et al. 2023c). Consequently, a novel high-throughput sequencing strategy should be used to identify genetic variants that are more accurately associated with flesh firmness at harvest.

In climacteric fruits such as apples, ethylene plays a pivotal role in fruit maturation. ACC oxidase 1 (*ACO1*) and ACC synthase 1 (*ACS1*) are located within a well-known QTL interval for fruit softening and are genetically linked to ethylene synthesis (Harada et al. 2000; Oraguzie et al*.*, 2004). Recently, a sery of allelic variations in ethylene response factors has been shown to be closely associated with firmness loss and ethylene production in apples (Hu et al. 2020; Wu et al. 2021b). However, insight into fruit softening before harvest remains largely unexplored. Because three traits (ripening date, firmness at harvest, and softening after harvest) are closely linked, systematic research is needed to elucidate this relationship.

During the development process of fruit, depolymerization of cell wall components and destruction of structure decrease cell adhesion and accelerate fruit softening (Brummell 2006). Moreover, cell walls often become nonexpandable, which makes expansins crucial since they positively participate in cell enlargement and cell wall creeping (Cosgrove 1997; Rose et al. 1997). Cell wall loosening during the ripening process is caused by synergistic modification of numerous cell wall hydrolases and non-enzymatic proteins known as expansins (EXPs). To date, knocking out only one cell wall-modifying gene other than pectate lyase does not fully explain the loss of firmness. Currently, expansins have been proposed as the main catalysts for breaking the frame construction formed by cellulose microfibrils and xyloglucan, thereby exposing polysaccharides to cell wall hydrolases (McQueen-Mason et al. 1992; Cosgrove, 2016).

EXPs are structural proteins involved in a variety of diverse biological processes, such as fruit ripening and softening (Costa et al*.* 2008; Perini et al. 2017; Mu et al. 2021) and flesh crispness retention (Costa and Stella, 2005; Trujillo et al. 2012). EXPs decrease the adhesion between adjacent cell wall polymers, as they directly mediate the creep of walls at low pH (Cosgrove 2000). Recently, EXP has been a hot research topic in the fruit softening process (Brummell et al. 1999; Valenzuela-Riffo and Morales-Quintana 2020). In tomatoes (*Solanum lycopersicum* L.), repression of *LeEXP1* resulted in reduced softening and delayed ripening, whereas overexpression of *LeEXP1* caused greater softening (Rose et al. 1997; Minoia et al. 2016). In apples, previous data suggested that varieties with a long shelf life have lower levels of *MdEXP3* expression during the maturation stage (Wakasa et al. 2003). However, the role of EXP in apples remains uncharacterized.

EXP, as a structural gene, is controlled by many upstream transcription factors (TFs) and hormone signals (Mu et al. 2021; Chen et al. 2024). To date, numerous TFs that mediate ripening and softening by modifying cell wall-related genes have been identified (Zhang et al. 2022b, 2023a). For example, the ethylene-related TF *MaERF11* delays maturation in bananas (*Musa* × *paradisiaca*) by inhibiting the transcription of *MaACO1* and *MaEXP* (Han et al. 2016). In maize (*Zea mays* L.), *ZmNAC29* enhances the transcriptional activity of *ZmEXPB15*, resulting in increases in kernel size and weight (Sun et al. 2022). In addition, it contains an abundant hormone-responsive element in the EXP promoter, which could participate in cell extension and cell wall modification regulated by various plant hormones, such as auxin-induced growth (Spartz et al. 2014; Cosgrove, 2016).

Our current understanding of ripening and softening relies on a few mutants in tomatoes, including non-ripening (NOR), ripening inhibitor (RIN), and colorless non-ripening (CNR) (Vrebalov et al. 2002; Manning et al. 2006; Gao et al. 2018). An increasing number of studies have shown that the NAC TF family plays a key role in regulating the ripening process in climacteric fruit. A number of NAC proteins from various species have been shown to be involved in ripening and quality traits formation by increasing the expression of genes associated with cell elongation, cell wall metabolism, and ethylene accumulation (Gao et al. 2020; Martín-Pizarro et al. 2021; Cao et al. 2023; Zhang et al. 2024).

Currently, data on EXP proteins and the flesh firmness of apples are still limited. Hence, a comprehensive understanding of the molecular regulation and genetic basis of this complex trait would provide potential insights into genetic modifications. Here, novel insights into firmness traits in apple fruit have been presented via the combination of BSA-seq, RNA-seq, and structural variation (SV) analysis. We functionally identified a candidate gene, *expansin-A1*, in apples, which is closely associated with flesh firmness. Additionally, a 1166 bp deletion in the promoter of *MdEXP-A1* decreases its transcription activity, which could be recognized and upregulated by an NAC TF. In addition, *MdNAC1* was also found to be capable of activating the expression of *MdEXP-A1*, which plays a positive regulatory role in ripening and softening.

## Results

### Segregation of flesh firmness at harvest

**Fig. 1:**
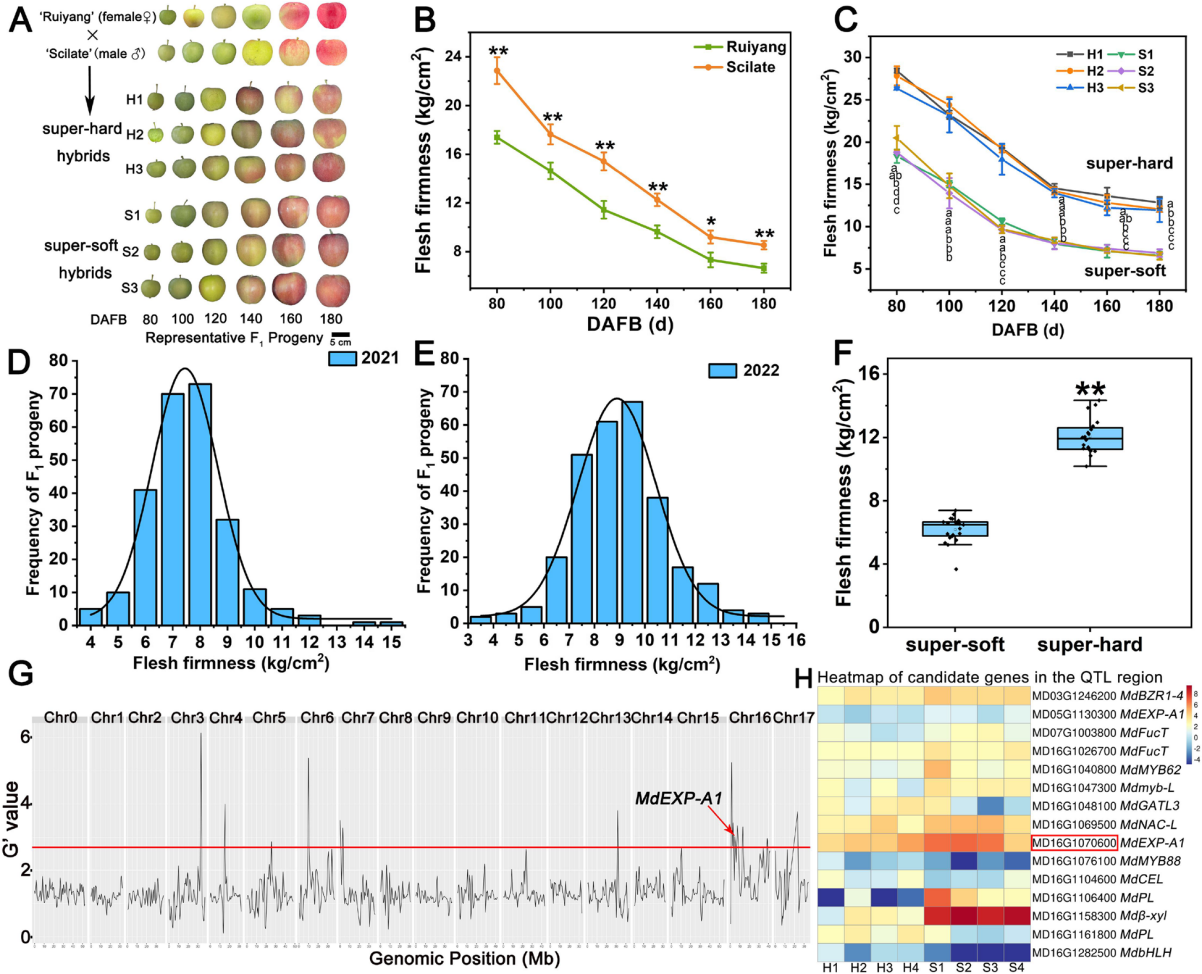
Phenotypic characterization and QTL identification for flesh firmness in the F_1_ population derived from ‘Ruiyang’ and ‘Scilate’. **A** Phenotypes of ‘Ruiyang’ and ‘Scilate’ and their super-hard and super-soft F_1_ individuals during diverse developmental periods. Images were digitally extracted for comparison. Scale bar =5 cm. **B** Flesh firmness changes in ‘Ruiyang’ and ‘Scilate’ apple fruits during various developmental stages. DAFB: days after full bloom. **C** Flesh firmness values of three super-hard hybrids (H1, H2, and H3) and three super-soft F_1_ hybrids (S1, S2, and S3) from F_1_ populations of apple fruit during different developmental stages. Different letters represent differences in the fruit development stages. From top to bottom, they are H1, H2, H3, S1, S2, and S3. Significance was defined at P < 0.05 (Student’s *t*-test). The frequency distribution of flesh firmness at harvest in the F_1_ population from a cross of ‘Ruiyang’ and ‘Scilate’ during 2021 (**D**) and 2022 (**E**). **F** The flesh firmness phenotype value of 20 super-hard and 20 super-soft hybrids for bulked segregant analysis (BSA) construction. **G** Identification of a candidate chromosome region for the flesh firmness trait at harvest via BSA-seq analysis in apples. **H** Heatmap of candidate cell wall genes and transcription factors within the QTL region for apple flesh firmness. The error bars represent the means ± SDs of three repetitions. Asterisks indicate a significant difference determined via a *t *test (*P <0.05, **P <0.01)

The ‘Scilate’ apple is popular worldwide for its hard-crisp texture, whereas the new cultivar ‘Ruiyang’ inherits the soft-crisp taste from its parent, ‘Fuji’. Throughout development, the firmness of ‘Scilate’ fruit was significantly greater than that of ‘Ruiyang’, and both the parents and representative progeny exhibited a dramatic decreasing trend (Fig. 1A-C). To understand the segregation regularity of flesh firmness, the phenotypic values of 251 F_1_ hybrids from ‘Ruiyang’ and ‘Scilate’ were collected at harvest for two consecutive years (Tables S1, S2). As a result, the firmness data exhibited broad segregation at harvest, and the major distribution range was 6–10 kg/cm^2^. The frequency distribution of firmness followed a normal distribution, suggesting that firmness at harvest is a multi-genic trait involving complex mechanisms (Fig. 1D, E). The broad-sense heritability (H^2^) was 84.81% and 91.36% in the two years for flesh firmness, respectively, indicating that the firmness trait at harvest was under strong genetic control in the offspring population (Table S3). The maximum firmness recorded was 14.18 kg/cm^2^, while the minimal value was 3.80 kg/cm^2^ (Table S3), indicating that the flesh firmness at harvest was widely segregated in the F_1_ population from ‘Ruiyang’ and ‘Scilate’, which could be utilized for QTL mapping analysis.

### Identification of *MdACS1* and *MdACO1* genotypes in F_1_ individuals

*MdACS1* and *MdACO1* are two well-known molecular marker genes associated with ethylene release and postharvest fruit softening. The genotypes of these two markers were identified via published primers (Harada et al. 2000; Oraguzie et al. 2004; Wen et al. 2024). The parental genotypes for ‘Ruiyang’ and ‘Scilate’ was *MdACS1-1/2:MdACO1-1/2* and *MdACS1-1/2:MdACO1-2/2* (Tables S4, S5). The homozygous *MdACS1* genotype was segregated at approximately 1:1 in F_1_ super-hard or super-soft hybrids (*MdACS1-2/2*:*MdACS1-1/1*), and the segregation ratio of *MdACO1* genotype was in a 1:1 ratio of the super-soft hybrids (Tables S4, S5). No significant effects were detected between the *MdACS1* or *MdACO1* genotype and flesh firmness at harvest in F_1_ hybrids from ‘Ruiyang’ and ‘Scilate’. Therefore, we still need to map more QTLs closely linked to flesh firmness at harvest.

### QTL analysis for flesh firmness in hybrids via BSA-seq

To uncover the key genetic basis and variation locus, two segregant bulks were constructed and re-sequenced from 20 super-hard and 20 super-soft individuals (Fig. 1F). Additionally, their parents were also re-sequenced. Clean reads for each sample (16,218,811,500 for super-hard pools and 16,755,700,200 for super-soft pools) were merged with the apple GDDH genome (Daccord et al. 2017). Candidate regions for flesh firmness at harvest were identified and located on Chr 03 (Chromosomes), 05, 06, 07, 13, and 16 from two segregant bulks (Fig. 1G, Fig. S1; Table 1). Among these QTLs, seven QTLs on Chr 03 overlapped at 33.2–33.3 Mb, and several QTLs were concentrated within an interval from 0‒17 Mb of Chr 16 (Table 1). The main QTL peaks were concentrated on five chromosome intervals (03, 06, 07, 13, and 16), and the G value was greater than the corresponding threshold value. The high enrichment variation of SNPs (Single nucleotide polymorphism) or InDels (insertion-deletion) in the above QTL regions may be positively associated with flesh firmness formation at harvest.

**Table 1:** QTL identification of apple flesh firmness characteristics

Chromosome	Start	End	Size(bp)	meanGprime	mean△SNP	N.SNP	N. Genes
Chr03	33,263,777	33,332,317	68,540	6.024101525	44	3	8
Chr04	15,390,046	15,390,046	0	3.994332499	Inf	1	0
Chr05	25,430,751	25,611,704	180,953	2.820156172	28	5	9
Chr06	9,187,563	10,546,221	1,358,658	3.727553504	13	17	29
Chr07	22,996	557,885	534,889	3.284809859	9	5	53
Chr07	2,396,448	3,017,503	621,055	2.996839436	6	4	78
Chr13	31,272,416	31,913,053	640,637	3.284236557	6	4	13
Chr16	1,446,291	3,664,502	2,218,211	3.297420478	9	20	313
Chr16	4,876,589	5,401,662	525,073	2.838387796	8	4	81
Chr16	6,740,198	8,624,692	1,884,494	2.991381221	44	83	238
Chr16	12,633,586	13,705,496	1,071,910	2.866823886	35	37	76
Chr16	38,239,603	38,958,525	718,922	2.833261878	40	29	24
Chr17	23,318,939	23,318,939	0	3.771079756	Inf	1	0

### Candidate gene screening for flesh firmness-associated QTLs at harvest

To narrow the research on the basis of flesh firmness genetics, all 922 genes were downloaded from the above QTL signal intervals. By combining previous transcriptome data of extra-hard and extra-soft hybrids (Su et al. 2024), a total of 109 DEGs were screened via a strategy with a |log2 (fold change)|> 1.0 and *P* values < 0.05 (Fig. S2; Table S6). According to the reference genome annotation, 9 candidate genes involved in cell wall modification and 6 transcription factors were identified and screened (Fig. 1H; Table S7). Furthermore, since fruit softening is well known to involve the disassembly of cell wall matrix polysaccharides, 9 candidate genes involved in cell wall modification were selected for further investigation. These genes included expansins (MD05G1130300 and MD16G1070600), O-fucosyltransferase family proteins (MD07G1003800 and MD16G1026700), galacturonosyltransferase (MD16G1048100), cellulase protein (MD16G1104600), pectate lyase (MD16G1106400 and MD16G1161800), and beta-xylosidase (MD16G1158300). Additionally, on the basis of previously published transcriptome data under fruit development (Liu et al. 2021; PRJNA728501), the transcript levels of these candidate genes during development were analyzed in detail (Fig. S3; Table S8). Finally, the expression level of cell wall-loosening gene (*MdEXP-A1*, MD16G1070600) and beta-xylosidase gene (MD16G1158300) increased significantly in three accessions during the whole development period, which was closely related to the decline of firmness. Therefore, these two genes were chosen for further study and verification.

### Analysis of genetic variation in candidate genes via Sanger sequencing

**Fig. 2:**
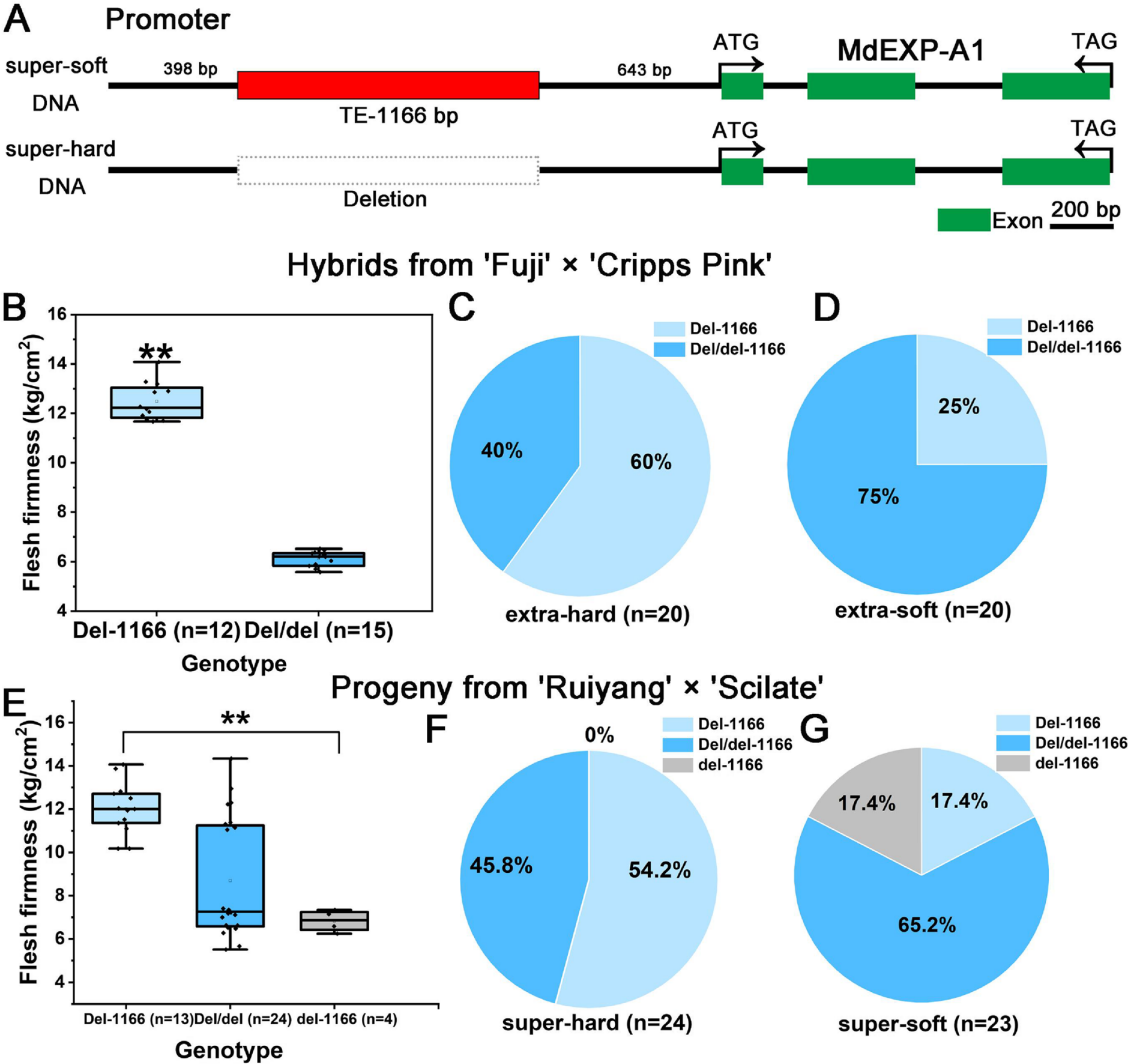
Identification and verification of a novel structural variation in F_1_ progeny from two crosses. **A** Gene structure and upstream genetic variation analysis of the candidate gene *MdEXP-A1*. **B** Correlation analysis of the flesh firmness at harvest in 20 extra-hard and 20 extra-soft hybrids of ‘Fuij’ and ‘Cripps pink’ and the SV loci genotypes. **C** Pie chart analysis of different genotypic ratios of this SV in the *MdEXP-A1* gene upstream in the 20 extra-hard individuals from ‘Fuij’ and ‘Cripps pink’. **D** Pie chart analysis of various genotypic ratios of this SV in the 20 extra-soft individuals from ‘Fuij’ and ‘Cripps pink’. **E** Correlation analysis of the flesh firmness phenotype in 47 super-hard and super-soft progeny of ‘Ruiyang’ and ‘Scilate’ and the SV locus genotypes. Pie chart analysis of various genotypic ratios of this SV in the 24 super-hard (**F**) and 23 super-soft individuals (**G**) from ‘Ruiyang’ and ‘Scilate’

To explore the genetic variation of key candidate genes, the coding sequence (CDS) and promoter of the candidate genes (MdEXP-A1, MD16G1070600 and Mdβ-xyl, MD16G1158300) were amplified from parents and linked into a 19-T vector for Sanger sequencing. As a result, the *MdEXP-A1* CDS in the parent was completely coincident with the ‘Golden Delicious’ reference genome (Fig. S4). However, upon comparison with the reference genome, 20 SNPs and two InDels in the promoter of *MdEXP-A1* were identified. Moreover, a novel structural variation (SV) of 1166 bp was absent in the promoter region of *MdEXP-A1* (Fig. 2A, Fig. S5 and S6). According to the website of the apple genome and CENSOR software, the SV loci were identified as transposable elements (TEs) that contain one LTR/BEL and one LTR/Gypsy family transposon (Fig. S7C). We used the 1166 bp sequence as a query to perform BLAST analysis on the NCBI database (https://blast.ncbi.nlm.nih.gov/Blast.cgi) and found that similar transposons were highly abundant across distinct chromosomes of the apple genome (Fig. S8). This allelic variation of *MdEXP-A1* was subsequently selected as a key genetic variation for further exploration.

### TE-1166 deletion in *MdEXP-A1* was associated with super-hard hybrids

To validate whether the SV loci was associated with flesh firmness at harvest, specific primers were designed via Primer 5 software (Table S11). The previous extra-hard and extra-soft F_1_ offspring (40 individuals) and their parents (Su et al. 2024), ‘Fuji’ and ‘Cripps Pink’, were subsequently genotyped via PCR (Table S9). Among the parents, the genotypes of ‘Fuji’ and ‘Cripps Pink’ were identified as heterozygous Del:del and homozygous deletion (Del-1166) from our PCR product analysis (Table S9). Interestingly, the Del-1166 genotype was found to be present in almost all extra-hard individuals, accounting for approximately 60% (12/20) (Fig. 2B, C). Conversely, this genotype was observed in only 25% of the extra-soft progeny (5/20) (Fig. 2D). Furthermore, 47 super-hard and super-soft F_1_ individuals from the cross of ‘Ruiyang’ and ‘Scilate’ were also genotyped. In the parents, the genotypes of the ‘Ruiyang’ and ‘Scilate’ accessions were identified as heterozygous Del:del (Table S10). Similarly, the Del-1166 genotype was closely linked to the super-hard hybrids, accounting for approximately 54.2% of the population (13/24) (Fig. 2E, F). In contrast, this genotype accounted for only 17.4% of the super-soft progeny (4/23) (Fig. 2G). In summary, the absence of the TE-1166 locus in the *MdEXP-A1* promoter was strongly correlated with the ultra-hard phenotype, and it could serve as a potential genotyping marker for assessing harvest firmness in apple MAS. Therefore, we speculated that the adjacent gene *MdEXP-A1* plays a crucial role in the firmness formation of apples because of its location within a significant QTL interval for flesh firmness, indicating its potential biological function as an essential candidate gene.

### Low expression of *MdEXP-A1* was closely related to the super-hard phenotype

**Fig. 3:**
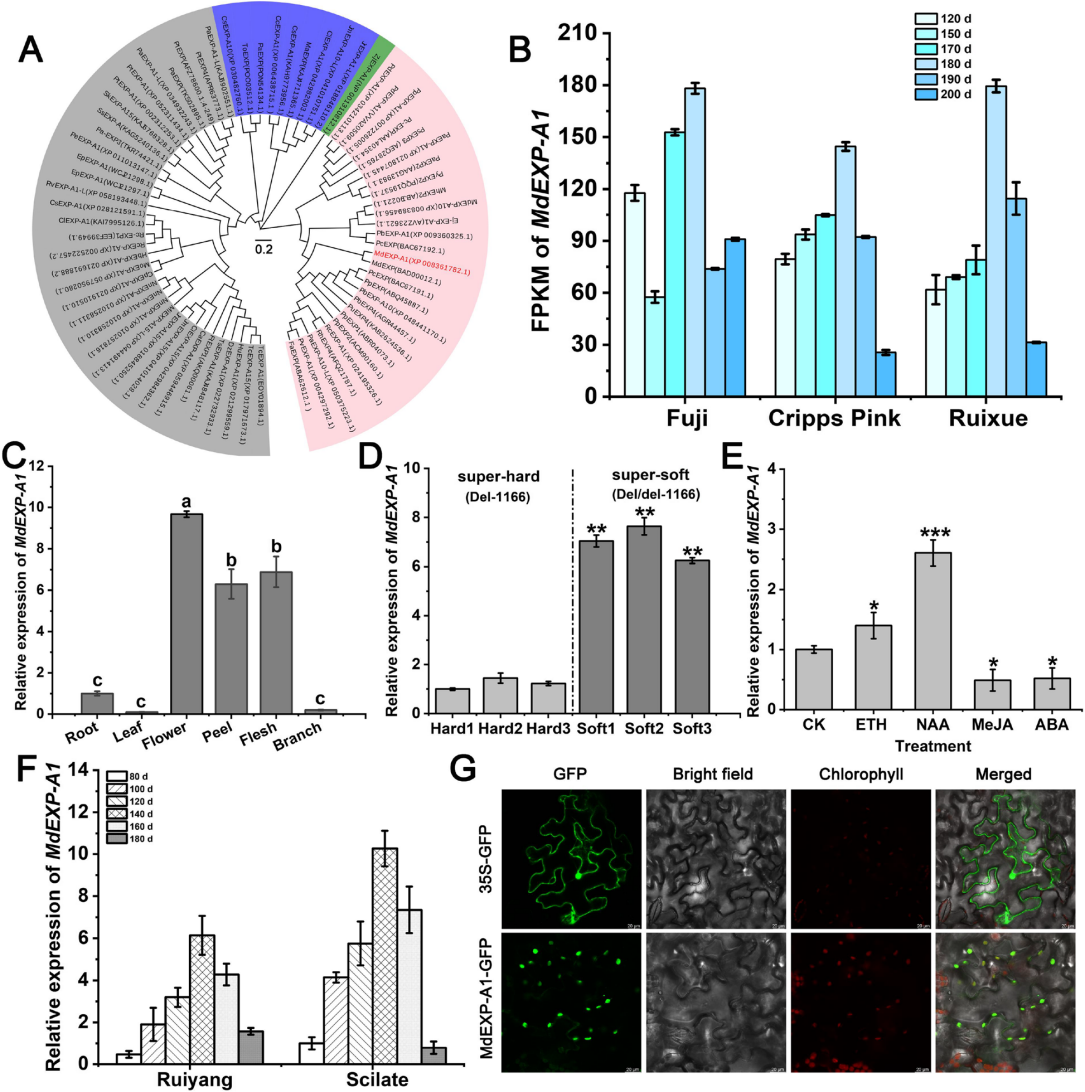
Identification of the *MdEXP-A1* gene correlated with flesh firmness. **A** Phylogenetic relationship analysis of *MdEXP-A1* in apples and orthologs in diverse species. **B** Expression patterns of *MdEXP-A1* in fruits of ‘Fuji’, ‘Cripps Pink’, and ‘Ruixue’ fruits during various developmental stages. **C** Relative expression analysis of *MdEXP-A1* in diverse apple tissues via RT‒qPCR. Different letters represent significant differences between diverse tissues. Significance was defined at *P* < 0.05 (Student’s *t*-test). **D** Transcription level of *MdEXP-A1* in super-hard (Del-1166) and super-soft (Del/del-1166) F_1_ hybrids at harvest period. **E** Expression level of *MdEXP-A1* in apple fruits after treatment with four hormones. **F** Relative expression level of* MdEXP-A1 *in the parents of ‘Ruiyang’ and ‘Scilate’ at various developmental stages. **G** Subcellular localization of the MdEXP-A1 protein. Scale bars = 20 μm. FPKM: Fragments per kilobase of exon model per million mapped fragments. The values are presented as the means ± SDs. Asterisks indicate a significant difference determined via a *t* test (*P <0.05, **P <0.01,***P <0.001)

Phylogenetic tree analysis highlighted the close relationship between MdEXP-A1 and other EXP proteins in the Rosaceae family (Fig. 3A). As indicated by previous transcriptome data under apple fruit development (Liu et al. 2021), this analysis revealed that the *MdEXP-A1* gene was present throughout the entire development process. The transcript of *MdEXP-A1* exhibited considerable accumulation during the development of the three apple varieties, and it was strongly expressed in both flowers and fruits according to RT‒qPCR (Reverse transcription-quantitative polymerase chain reaction), indicating that the expression of *MdEXP-A1* was highly positively correlated with a decrease in firmness during development (Fig. 3B, C). Additionally, the results revealed that the expression of *MdEXP-A1* was significantly lower in three super-hard F_1_ individuals (4J-11, 4J-63, and 4J-154) with the Del-1166 genotype than in the super-soft population (4J-112, 4J-103, and 4J-130) with the Del/del genotype at harvest (Fig. 3D). Additionally, after the fruits were sprayed with the four hormones, the transcript level of *MdEXP-A1* was strongly elevated in the NAA treatment group compared with the control group, indicating that the expression of* MdEXP-A1* was potentially regulated by the auxin signaling pathway (Fig. 3E). Similarly, its transcriptional level reached its peak at the fruit expansion stage during the development of the parents, ‘Ruiyang’ and ‘Scilate’, supporting its potential role in regulating cell enlargement and cell wall extensibility during development (Fig. 3F). Overall, we propose that *MdEXP-A1* plays an active role in the development stage. In addition, the subcellular localization of the MdEXP-A1 protein was located in the chloroplast, whereas the 35S::GFP was distributed throughout the cell, which may be related to cytoplasmic inheritance (Fig. 3G). As a result, *MdEXP-A1* was subjected to further functional validation.

### *MdEXP-A1* promotes fruit softening in ‘Fuji’ apples

**Fig. 4:**
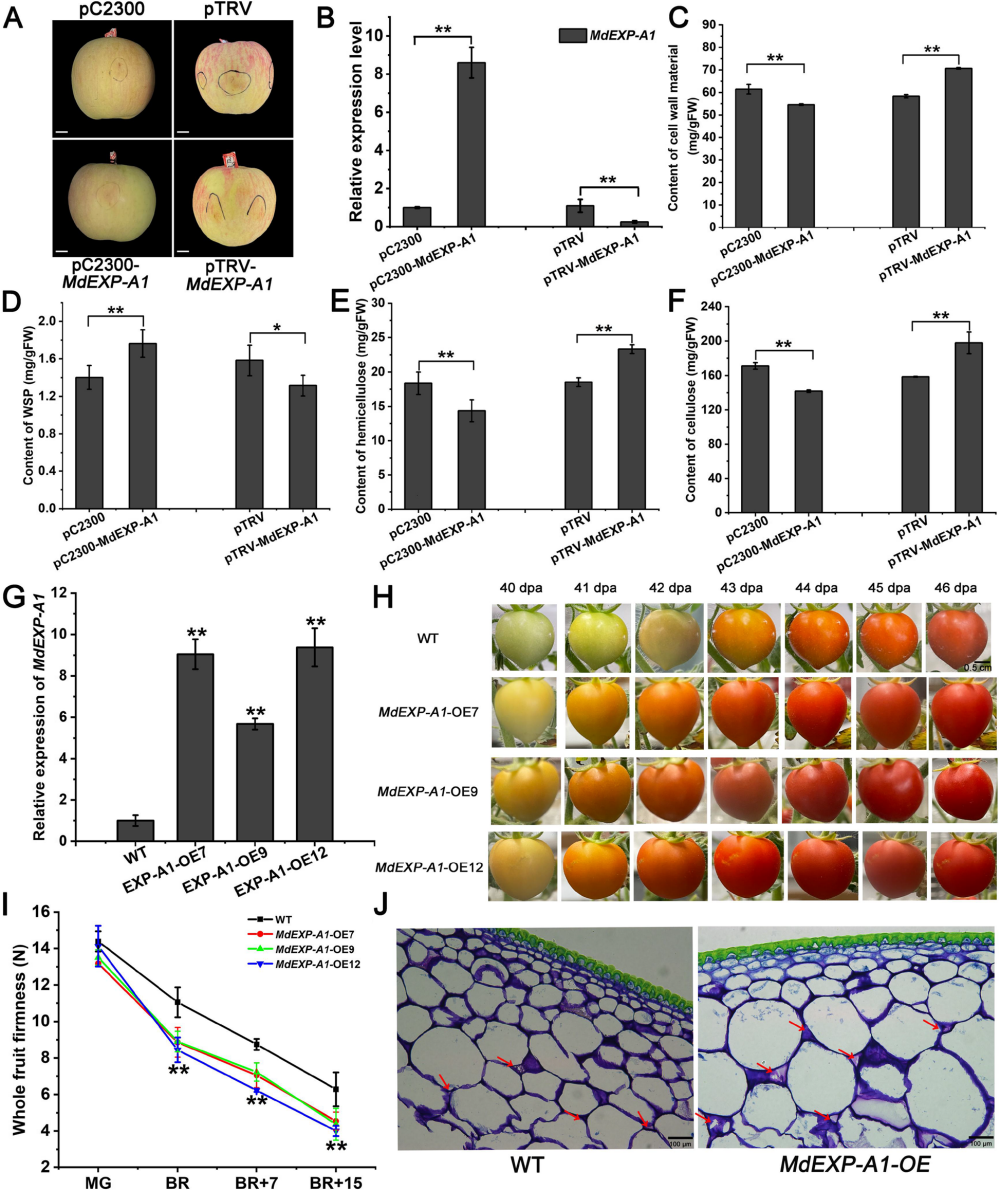
*MdEXP-A1* positively facilitates flesh softening in apples and tomatoes. **A** Transient overexpression and silent expression of *MdEXP-A1* in ‘Fuji’ apple fruits. Images were digitally extracted for comparison. Scale bar = 1 cm. **B **Relative expression levels of *MdEXP-A1* in apple fruits after transient injection. **C** Determination of fruit cell wall material after transient expression of *MdEXP-A1*. Cell wall component content of WSP (**D**), hemicellulose (**E**), and cellulose (**F**) in instantaneously injected apple fruits. **G** The relative expression of the *MdEXP-A1* gene in break-stage fruits of three independent over-expression lines. **H** Phenotypic observation of WT and *MdEXP-A1*-OE lines at diverse stages of fruit development and ripening phases. The fruits from three independent transgenic tomatoes showed an early veraison phenotype. Scale bar = 0.5 cm. **I** Firmness changes in *MdEXP-A1* transgenic tomato lines and wild-type tomatoes during development and post-harvest stages. **J** Fruit cell structure of *MdEXP-A1* transgenic tomato lines and wild-type during the Break+7 stage. Scale bar = 100 μm. The error bar represents the standard error of 3 replicates. Asterisks indicate a significant difference determined via a *t*-test (*P <0.05, **P <0.01)

The EXP-A1 protein of nine species was highly conserved and may have similar functions (Fig. S9). To explore the potential function of *MdEXP-A1* in firmness formation, we first cloned the CDS of *MdEXP-A1* from ‘Golden Delicious’. Next, the *MdEXP-A1-*pCAMBIA2300 recombinant plasmids were used for transient overexpression in ‘Fuji’ apple fruits. After being placed in the dark for five days, a remarkable increase in *MdEXP-A1* expression and the content of water-soluble pectin (WSP) and a dramatic reduction in the content of cell wall materials, hemicellulose, and cellulose were detected in fruits transiently injected with *MdEXP-A1-*pCAMBIA2300 compared with those injected with the empty vector (Fig. 4A-F). Moreover, the VIGS (virus-induced gene silencing) technique was used to further verify the effects of *MdEXP-A1* on flesh firmness. Compared with those in ‘Fuji’ apples infected with a mixture of pTRV2 and pTRV1 vectors, the expression levels of *MdEXP-A1* and WSP content in pTRV2-*MdEXP-A1*-transformed apple fruits were significantly lower after five days of infiltration (Fig. 4B, D). The contents of cell wall material, hemicellulose, and cellulose after silencing by *MdEXP-A1* were significantly higher than those after being injected with the empty vector (Fig. 4C, E, and F). These data indicate that *MdEXP-A1* can promote apple flesh softening by disrupting pulp cell adhesion and the polysaccharide structure of the cell wall.

### *MdEXP-A1* promotes early color breakdown in transgenic tomato

Given that tomatoes serve as a classic model for the genetic studies of fruit development and ripening, we overexpressed *MdEXP-A1* in ‘Micro Tom’. Several independent transgenic lines of *MdEXP-A1* were obtained via the leaf disc method. Furthermore, they were identified via PCR and RT‒qPCR. The amplified target DNA band and increasing transcript level indicated successful overexpression of *MdEXP-A1* in tomato (Fig. 4G; Fig. S10). Compared with those of wild-type (WT) fruits, the fruits of three stable T3 generation seedlings whose expression was high presented accelerated ripening (Fig. 4H). Additionally, no significant difference in flesh firmness was detected at the mature green (MG) stage, whereas the overexpression of *MdEXP-A1* led to softer tomato fruits, especially at the BR, B7, and B15 stages (Fig. 4I). Our cytological observations revealed that the cell gap of the transgenic fruits was greater than that of the WT fruit at the B7 stage (Fig. 4J). These results demonstrated that the candidate gene *MdEXP-A1* was responsible for firmness formation during development.

### The deletion of TE-1166 in the *MdEXP-A1* promoter reduces its transcription activity

**Fig. 5:**
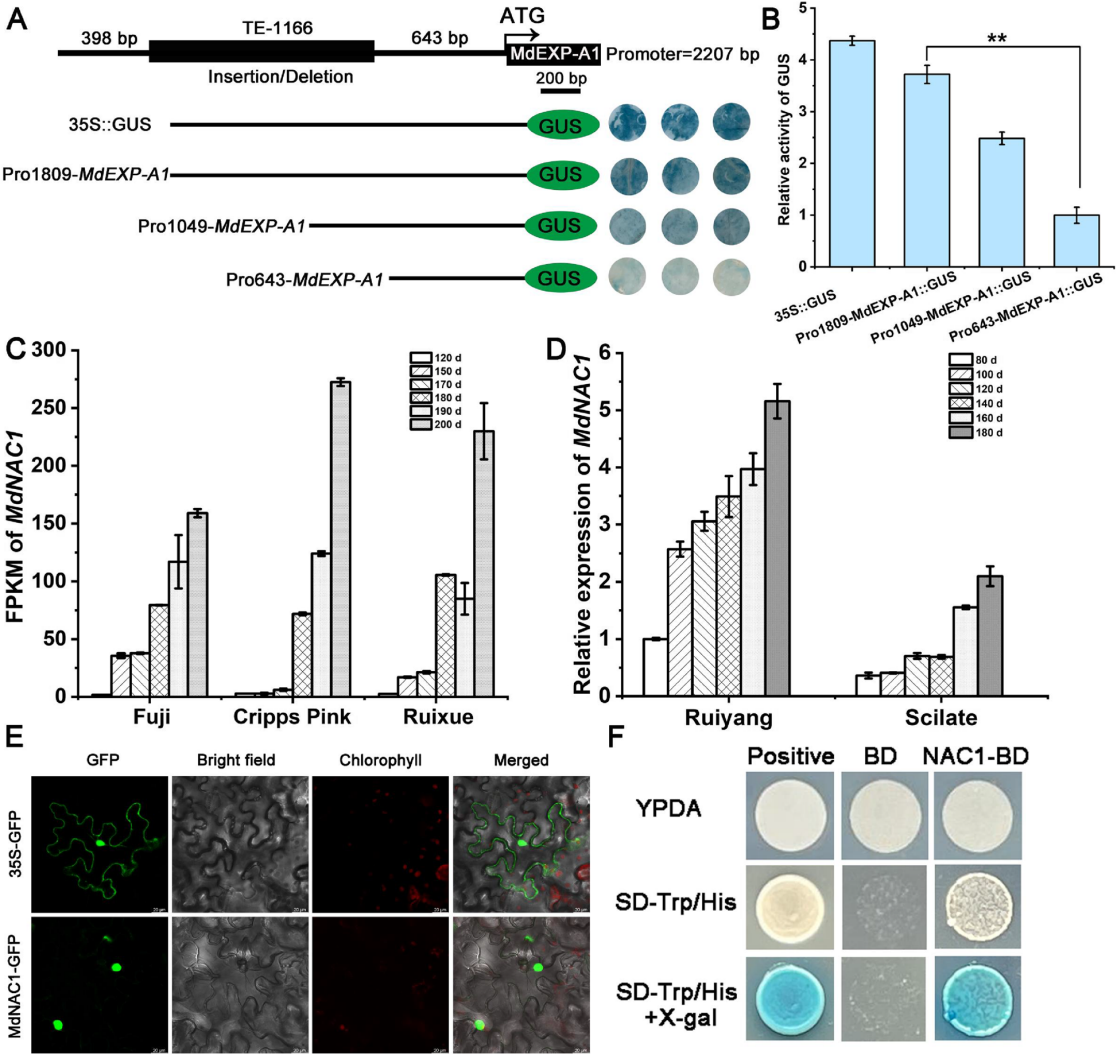
Transcriptional activity analysis of the structural variation and identification of a regulatory transcription factor, *MdNAC1*. **A** B-glucuronidase (GUS) staining of the truncated fragment of the *MdEXP-A1* promoter in transgenic tobacco leaves. **B** The relative activity of GUS in the truncated promoter of *MdEXP-A1*. **C** Transcript accumulation of *MdNAC1* in ‘Fuji’, ‘Cripps Pink’, and ‘Ruixue’ fruits during development. **D** Expression patterns of *MdNAC1* in parents of ‘Ruiyang’ and ‘Scilate’ fruits throughout the entire developmental stage. **E** Subcellular localization signal of the MdNAC1 protein in tobacco leaves. Scale bars = 20 μm. **F** Transcriptional activation activity analysis of the MdNAC1 protein in the yeast system. The error bars represent the standard error of 3 replicates. Asterisks indicate a significant difference determined via a *t* test (**P <0.01)

Owing to the SV loci occurring in the promoter region of *MdEXP-A1*, the specific 1166 bp sequence was analyzed in detail according to the PlantCARE website (https://bioinformatics.psb.ugent.be/webtools/plantcare/html/). As a result, a series of *cis*-acting elements that can be recognized by hormones, light signals and multiple TFs, such as MYC, ERF, and NAC, were identified (Fig. S11). In addition, the SV loci contain multiple CAAT boxes, which play a strong regulatory role in enhancing promoter activity and increasing gene transcription levels. Moreover, many *cis*-acting elements of the full-length promoter (2207 bp) were also identified (Fig. S12). To further investigate whether this SV affects its transcriptional activity, the promoter of *MdEXP-A1* was amplified via segmentation and independently linked into the pCAMBIA1301 vector with 35S removed. Tobacco leaves were subjected to transient transformation via *Agrobacterium* injection. As shown in Fig. 5A, transformants of pro1829-*MdEXP-A1*::GUS (containing TE-1166) presented greater GUS staining intensity than did those of pro643-*MdEXP-A1*::GUS (lacing TE-1166). These data suggested that the TE-1166 deletion of the *MdEXP-A1* promoter could reduce its transcription activity (Fig. 5A, B).

#### MdNAC1 binds the *MdEXP-A1* promoter and promotes its transcriptional activity

**Fig. 6:**
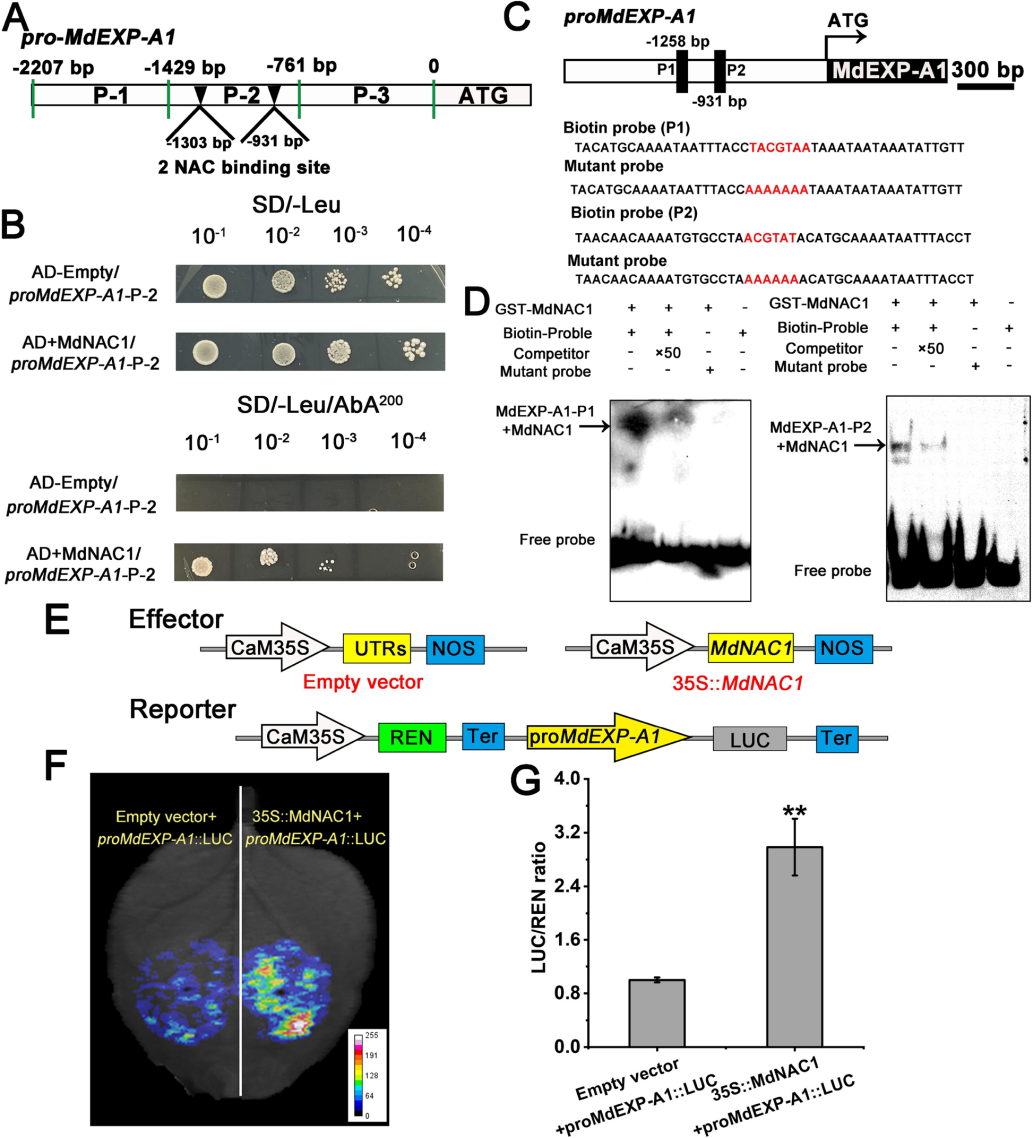
MdNAC1 binds the *MdEXP-A1* promoter and promotes its transcriptional activity. **A** Location analysis of NAC-binding *cis*-acting elements in the *MdEXP-A1* promoter sequence. **B** Yeast one-hybrid assay results showing showed that MdNAC1 can interact with the P-2 fragment of the *MdEXP-A1* promoter, and the empty vector (AD) was used as a negative control. **C** The probe in the *MdEXP-A1* promoter sequence, the binding probe, is a biotin-labeled fragment containing an [ACG] motif. The cold probe is an unlabeled competitive probe (50 times the concentration of the binding probe). The mutation probe contains seven or six nucleotide substitutions. **D** Electrophoretic mobility shift assays revealed that MdNAC1 binds to the specific motifs P1 and P2 of the *MdEXP-A1* promoter. The ‘ + ’ and ‘-’ represent the presence and absence of the probe or protein, respectively. **E** Constructs used to test the role of *MdNAC1* in *MdEXP-A1* expression in transient expression assays. **F** and **G** LUC assay indicating that MdNAC1 positively promotes the activity of the *MdEXP-A1* promoter. The values are the means ± SDs of three independent biological replicates; Asterisks indicate significant differences determined via a *t* test (**P < 0.01)

Owing to the absence of a large fragment on the *MdEXP-A1* promoter, it was speculated that transcriptional regulation is one of the major causes of the different expression patterns of *MdEXP-A1* in super-hard and super-soft hybrids. Additionally, several *cis*-regulatory elements of the SV have been explored, and two NAC-specific binding motifs (AC GTA) have been discovered (Zhong et al. 2010; Nieuwenhuizen et al. 2015). Numerous studies have shown that the NAC protein is a strong predictor of maturity and softening (Yeats et al*.* 2019; Cao et al. 2021). Next, we identified an NAC TF called *MdNAC1*, which is involved in the regulation of pigment accumulation (Liu et al*.* 2023). Furthermore, the expression of *MdNAC1* was assessed in both parents and three cultivars during development (Fig. 5C, D). These results indicate a significant increase in its transcriptional level in ‘Ruiyang’, ‘Scilate’ and other varieties throughout development, supporting its potential role in ripening and softening. The MdNAC1 protein subsequently localized to the cell nucleus with transcriptional activation activity in the yeast system (Fig. 5E, F), suggesting its typical TF characteristics. To validate whether *MdNAC1* can bind to the deleted promoter of *MdEXP-A1*, the fragment (P‒2) of the promoter was inserted into the pAbiA vector for yeast one-hybrid analysis. The result indicated that *MdNAC1* can interact with the deletion promoter fragment of *MdEXP-A1* (Fig. 6A, B). Furthermore, two specific probe primers were designed for the EMSA (electrophoretic mobility shift assay) experiment, and the results again revealed that *MdNAC1* can bind these two sites (Fig. 6C, D). To explore the effect of binding on *MdEXP-A1* transcription, a luminescence (LUC) assay involving genetic transformation of tobacco was performed. The LUC intensity results indicated that *MdNAC1* can increase the expression of *MdEXP-A1* (Fig. 6E–G). In conclusion, our findings revealed that *MdNAC1* could bind to the promoter fragment and activate the transcription of *MdEXP-A1*.

#### MdNAC1 expedites fruit ripening and softening

**Fig. 7:**
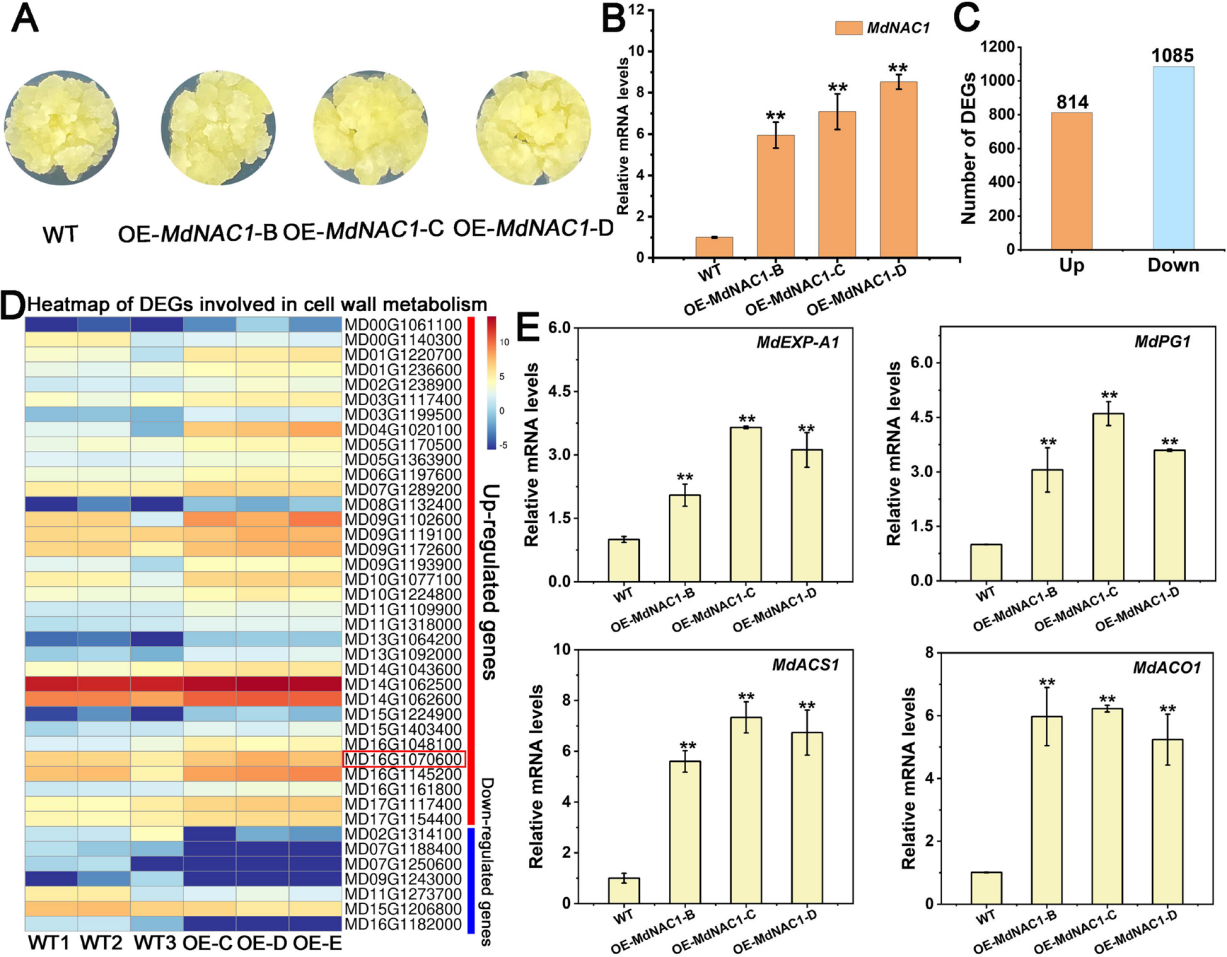
Overexpression of *MdNAC1* in stable transgenic apple calli. **A** Phenotype of *MdNAC1* stable overexpressing ‘Orin’ calli lines. **B** The relative expression levels of the *MdNAC1* gene in transgenic apple calli were determined via RT‒qPCR. **C** The number of differentially expression genes (DEGs) between WT and *MdNAC1* transgenic calli. up: up-regulated; down: down-regulated.** D** Heatmap of all DEGs involved in cell wall metabolism from WT and stable *MdNAC1* transgenic calli lines. **E** Transcription levels of firmness-related genes in WT and 35S::*MdNAC1* calli

**Fig. 8:**
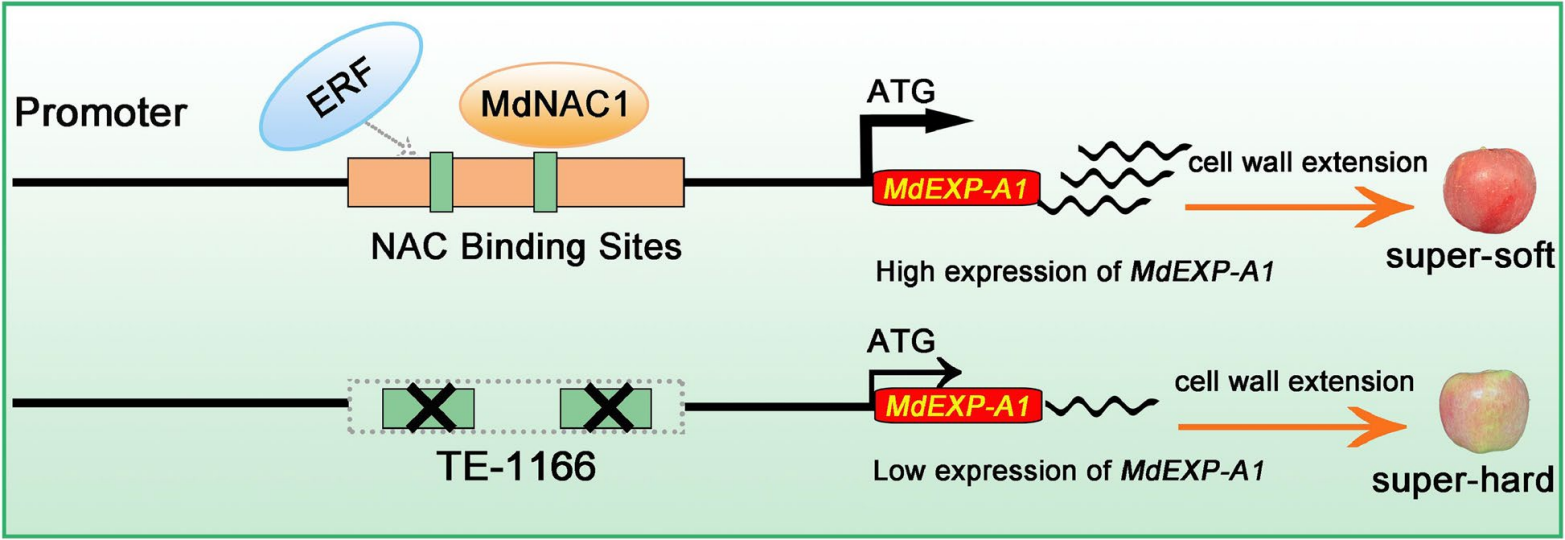
A proposed model for illustrating the effects of the TE-1166 and MdNAC1 on the expression of *MdEXP-A1* in regulating apple flesh firmness. The thin and thick lines with arrows indicate low and high expression of *MdEXP-A1*, respectively. The wavy line indicates the abundance of MdEXP-A1 expression protein. TE-1166 can be recognized and upregulated by the upstream regulatory factor MdNAC1, resulting in super-soft-fleshed hybrids. The dotted arrows indicate that the mechanism of ERF on *MdEXP-A1* is unknown

*MdNAC1* can increase the transcription level of *MdEXP-A1*, which may play a positive regulatory role in the ripening and softening of fruit. Three independent *MdNAC1*-overexpressing lines were subsequently obtained from ‘Orin’ apple calli. The positive transgenic calli lines were subsequently verified via PCR and RT‒qPCR (Fig. 7A, Fig. S13). Furthermore, by using |log2FC|> = 1 and q < 0.05 as the criteria, a total of 1899 DEGs were identified via RNA-seq analysis of *MdNAC1* transgenic lines and WT calli (Fig. 7C, Fig. S14A). GO analysis revealed that those DEGs were enriched in cell wall metabolism, plant hormone signaling, and the defense response pathway (Fig. S14D). Compared with those in the control lines, 34 cell wall-related DEGs, including *MdEXP-A1* gene, were upregulated and 7 were downregulated (Fig. 7D, highlighted in red box). The expression of the firmness-related marker genes *MdACS1*, *MdACO1*, and *MdPG1* was obviously elevated, supporting its regulatory role in ethylene synthesis and cell wall metabolism. As expected, the relative expression of the *MdEXP-A1* gene was significantly greater in the *MdNAC1*-overexpressing lines than in the control calli (Fig. 7E). Combined in vitro and in vivo experiments provided novel insight into the regulation of flesh firmness formation by demonstrating the involvement of *MdNAC1* in the modulation of the deleted fragment of *MdEXP-A1*. In summary, the above studies demonstrated that the presence of TE-1166 can be recognized by upstream MdNAC1 transcription factor and promote the expression of the downstream gene *MdEXP-A1*, which is involved in the formation of flesh firmness at harvest (Fig. 8).

## Discussion

Flesh firmness at harvest is a major objective breeding trait worldwide. In the practical production of apples, flesh firmness is commonly used as an indicator of ripeness. In general, early-maturing cultivars tend to have shorter shelf life (Kenis et al. 2008). Additionally, for one cultivar, higher firmness at harvest leads to a longer shelf-life and highlights the tight genetic linkage in those traits. Nevertheless, the underlying mechanisms remain elusive. Owing to the high heterozygosity of apple, the key challenge in determining the genetic mechanism of flesh firmness is to identify major genes and functional variants.

A total of 251 hybrids from ‘Scilate’ and ‘Ruiyang’ were investigated for flesh firmness at harvest over two years. These populations exhibited extensive segregation (Fig. 1D, E), suggesting that flesh firmness is a typical quantitative trait controlled by multiple genes. Next, we combined BSA-seq and QTL analysis to identify major genetic loci mapped, especially to Chr03, Chr06, Chr07, and Chr16. In previous studies, numerous QTLs and candidate genes associated with apple fruit softening have been identified. For example, *NAC18.1* was characterized within a major QTL on Chr03 for apple maturity date, and flesh firmness at harvest variation on its CDS led to various harvest dates (Migicovsky et al. 2016; Yeats et al. 2019). In addition, a series of natural variations in apple ERF TFs have been revealed on Chr03 and Chr16, which are responsible for ethylene production and firmness loss (Hu et al. 2020; Wu et al. 2021b). Those studies indicated that Chr03 and Chr16 were hotspot intervals of flesh firmness and postharvest softening.

To date, abundant genetic variations have been progressively elucidated and converted into molecular markers for firmness and shelf-life prediction, especially in *MdACS1* and *MdACO1* (Costa et al. 2005; Oraguzie et al. 2007; Costa et al. 2010). In our study, there was no significant correlation between the *MdACS1* and *MdACO1* genotypes and firmness diversity in the populations of ‘Ruiyang’ and ‘Scilate’. It is possible that *MdACS1* or *MdACO1* may function only in certain combinations of parental genotypes. Additionally, a growing body of research has reported that the predictive power of these functional markers is limited in terms of phenotype diversity (Nybom et al. 2013; Wu et al. 2021a), suggesting that many other genes and variants that may contribute to firmness change. These findings further indicate that the mechanisms of flesh firmness at harvest and postharvest softening vary, which is in agreement with previous research (Wu et al. 2021b).

Through multi-omics analysis of BSA and comparative transcriptome sequencing in extra-hard and extra-soft hybrids, nine cell wall-associated candidate genes within the QTL region were screened. In addition, combined with the transcriptome data under fruit development previously published by Liu et al. (2021), the EXP and beta-xylosidase candidate genes with significantly increased expression at a major QTL interval (Chr16) were selected for further study. With the development of high-throughput re-sequencing technologies, many studies have revealed that large SVs play essential roles in the adaptive evolution of species and key trait formation (Zhang et al. 2019; Li et al. 2023a). Next, a comparison of the results of genome analysis by next-generation re-sequencing and Sanger sequencing helped identify a novel SV in the promoter of *MdEXP-A1* (Fig. S5, S6). Furthermore, the genotype of this TE-1166 was validated in F_1_ super-hard and super-soft populations, and the results revealed that homozygous deletion of this SV was strongly associated with the super-hard phenotype at harvest, which could be a potential diagnostic marker for the firmness trait of apple MAS application. Together, the evidence again suggested that the adjacent gene *MdEXP-A1* of this variant was closely linked to hardness formation at harvest.

As mentioned above, the MdEXP-A1 proteins are highly conserved in diverse species, indicating that they may perform similar biological functions. EXP is a non-enzymatic cell wall loosening protein that participates in cell enlargement and cell wall creep, which is positively associated with fruit ripening and softening (Cosgrove 1997; 2016; Rose et al. 1997). For example, *MdEXP7* was mapped on Chr01 in a major QTL interval, and its allelic variation in the promoter was associated with fruit softening (Costa et al., 2008). Owing to the extension and expansion of the cell wall during development, the cell turgor and fruit firmness decreased sharply, and the intercellular space increased, so the EXP protein was proposed to be associated with changes in flesh texture. In this study, a candidate EXP gene was selected from a multi-omics conjoint analysis. As the fruit ripens, the transcript level of *MdEXP-A1* in the parents peaks during the expansion stage, and its transcript level decrease after plung into ripening (Fig. 3F). These results are consistent with previous reports on apples (Wakasa et al. 2003) and pears (Hiwasa et al. 2003), supporting the potential role of fleshy fruits during development.

EXP crude protein was first extracted from cucumber seedlings and is well known to induce the extension and relaxation of cell walls via the ‘acid growth’ mechanism (McQueen-Mason et al. 1992; Cosgrove, 2016). According to our earlier study, flesh malic acid sharply decreases throughout development (Su et al. 2022). Therefore, we speculated that its expression activity was inhibited at harvest. Additionally, preharvest NAA treatment promotes the expression of *MdEXP-A1*, which is consistent with their role in the response of cell elongation to auxin-induced acidification (Cosgrove, 2016), but the in-depth mechanism of their interaction requires further exploration. In our study, transient overexpression of *MdEXP-A1* clearly disrupted the cell wall polysaccharide matrix structure of apple, and ectopic expression of *MdEXP-A1* facilitates the ripening and softening of tomato fruits (Fig. 4), which have functions similar to those of *SlEXP1* in tomatoes (Brummell et al. 1999). Cell wall disassembly during the softening process is caused by the coordinated action of cell wall hydrolases and expansin (Brummell and Harpster 2001; Brummell 2006). A two-step model system for fruit softening in which expansins act in the expansion stage and cell wall polysaccharide are subsequently degraded by cell wall enzymes has been proposed. Inhibition of *EXP1* gene alone had no significantly effect on fruit firmness in tomatoes. A recent study demonstrated that *SlExp1* and *SlCel2* coordinate to regulate the decline in tomato firmness (Su et al. 2023). Overall, flesh firmness is a multigenic trait associated with cell wall metabolism.

On the basis of our variant validation, the absence of TE-1166 in the *MdEXP-A1* promoter was obviously correlated with super-hard hybrids at harvest. In addition, the expression of *MdEXP-A1* in the super-hard offspring with Del-1166 was noteworthy lower than that in the super-soft populations of the Del:del genotype (Fig. 3D). Furthermore, the presence of Del-1166 increased its transcriptional activity (Fig. 5A, B). We speculated that the existence of this SV may facilitate the activity of the MdEXP-A1 protein. In addition, this variation location could be recognized by several TFs, such as MYC, ERF, and NAC (Fig. S11). In particular, NAC has been proposed as a strong determinant of fruit harvest date and firmness-related traits (Yeats et al., 2019). These findings suggested that the variant site of *MdEXP-A1* may be subject to specific transcriptional regulation. These results again indicate that the MdEXP-A1 is a major functional protein affecting flesh firmness and that its allelic variation may play a key role in the evolution and domestication of apple fruits.

The highly conserved function of the NAC orthologs in diverse species has been proven to play an active regulatory role in ripening and softening (Zhang et al. 2024). What’s more, the expression level of *MdNAC1* trended to increase during development in multiple varieties and exhibited transcriptional activation activity (Fig. 5C, D, and F). These results may support its critical effect on the ripening pathway and downstream structural gene regulation. In our study, the upregulated DEGs from the stable overexpression lines suggested that *MdNAC1* has multiple positive effects on fruit ripening and softening (Fig. 7C, D). However, it cannot be excluded that other apple NAC members and TFs play similar roles in apple maturity. Our study integrated EMSA, Y1H (yeast one-hybrid), Dual-Luciferase, and stable overexpression assays and revealed that *MdNAC1* upregulates the *MdEXP-A1* mechanism for flesh firmness. This finding is in agreement with a previous study in which the expression of EXP was inhibited in NOR mutants (Gao et al. 2018). Furthermore, the expression of *MdACS1* and *MdACO1* was significantly elevated in the *MdNAC1* transgenic calli. These results demonstrated that *MdNAC1* may promote the ripening and softening of apples by controlling ethylene production and cell wall creeping. Recently, reports have revealed strong similarities in the modification of NAC homologs in tomato (Gao et al. 2020), strawberry (Li et al. 2023b), banana (Shan et al. 2012), and peach (Zhou et al. 2023; Zhang et al. 2024). Additionally, the NAC protein participates in the regulation of quality formation by modifying specific structural genes; therefore, investigating distinct genetic networks to balance ripening and quality is crucial for breeding programs (Cao et al. 2023).

## Materials and methods

### Plant materials and growth conditions

A total of 251 F_1_ individuals from a cross of ‘Ruiyang’ and ‘Scilate’ were planted on M26 rootstock at the experimental station of Northwest A&F University, Baishui County, Shaanxi Province, China. Both parents and super-hard or super-soft progeny were sampled individually from 80 days after full bloom (DAFB) until ripening, with a 20-day interval. The root, leaf, flower, pericarp, pulp, and branch tissues were collected from the ‘Fuji’ cultivar. The management level of the orchard was coincidental for all apple offspring. Apple fruits were chosen according to taste, base color, days after full bloom, and degree of starch clearance (starch value of 7). Three biological replicates of 4–6 fruits per replicate were selected for detection.

### Exogenous hormone treatment

One hundred fruits of ‘Ruixue’ apple (each twenty) were sprayed with 0.5 mM jasmonic acid methylester (MeJA), 0.5 g/L ethylene (ETH), 4 mM naphthylacetic acid (NAA), or 0.4 g/L abscisic acid (ABA) two weeks before harvest, and water spray was used as a control. The surface of the apple fruits was sprayed with the above hormone mixture until it had dripped down. The fruits were sprayed again after 7 days and finally picked at harvest.

### Determination of flesh firmness

The firmness of all the apple samples was assessed at opposite sides of the equatorial portion of the peeled fruit with a texture analyzer (GS-15, Germany). The flat probe has a diameter of 10 mm and a test depth of 8 mm. Alternatively, the hardness of the tomatoes was determined by another texture analyzer (TA-XT plus, UK) with a 2 mm diameter probe (1.5 mm/sec, depth: 8 mm). Three biological replicates were performed, with six fruits as one replicate.

### BSA-seq and QTL identification

On the basis of firmness data from the hybrids for two consecutive years, a total of 40 individuals with super-hard or super-soft phenotypes were selected to establish two pools. Young leaves of representative hybrids or parents of ‘Ruiyang’ and ‘Scilate’ were used to extract total genomic DNA. Next, two bulked libraries were generated on the basis of the DNA equivalence principle. These libraries were subsequently sequenced via the Illumina sequencing platform, and 150 bp paired-end reads were obtained. Burrows-Wheeler Aligner (BWA) was used to align the processed reads of each sample against the reference genome. SNP/InDel detection and annotation were performed via GATK and ANNOVAR software. The Δ (SNP index) and G values analysis between the super-hard pool and the super-soft pool were subsequently calculated to obtain a candidate region correlated with flesh firmness at harvest.

### Candidate gene prediction on the basis of BSA-seq and RNA-seq data

QTL identification of firmness at harvest was narrowed down to special regions by BSA analysis. Using combined transcriptome data from previous individuals with an extreme firmness phenotype or during development, several key candidate genes were subsequently screened to explore genetic variation and function. Briefly, all genes were downloaded from the QTL regions for flesh firmness at harvest, and DEGs were further screened from previous RNA-seq data. According to the annotation of the apple genome, genes associated with cell wall metabolism were chosen as candidate genes for flesh firmness trait. The transcript levels of candidate genes during development were subsequently analyzed via previously published data (Liu et al. 2021).

### Extraction of genomic DNA and RNA, and real-time qPCR analysis

The genomic DNA of representative F_1_ hybrids and parents, tomatoes, and apple calli lines was generated with a plant DNA kit, and the total RNA of apple, tomato, and calli samples was extracted using a plant quick RNA kit (Tiangen, Beijing, China). Then, first-strand cDNA and RT‒qPCR were conducted as previously described (Su et al. 2022). *Mdactin* (MD01G1001600) was used as the internal reference. The primers used for RT‒qPCR are listed in Table S11.

### Discovery and validation of genetic variation

The CDSs and promoters of the candidate genes were amplified and sequenced via Sanger sequencing. The design of specific primers for the *MdEXP-A1* promoter was based on the reference genome (Table S11). The genotypes of the F_1_ hybrids were identified by corresponding primers for *MdACS1* and *MdACO1* as previously described (Harada et al. 2000; Costa et al. 2005; Wen et al. 2024). The PCR system for this genetic structure of *MdEXP-A1* was performed as previously reported by Ma et al. (2021).

### Subcellular localization analysis

The recombinant proteins of MdEXP-A1 and MdNAC1 with a GFP label were ligated into the pCAMBIA2300 vector, and the corresponding primers used are shown in Table S11. The empty vector and each recombinant plasmid were individually transformed into *Agrobacteriun* strains (GV3101), after which they were separately infected into tobacco leaves (one month old). After three days of co-expression, GFP images were acquired via a confocal laser scanning microscope at an excitation wavelength of 488 nm (Leica TCS-SP8 SR).

### Transcriptional activity assays

The transcriptional activity assay was performed as described by Wang et al. (2023a). The *MdNAC1* CDS, lacking a termination codon, was constructed into the pGBKT7 carrier with a BD domain. The primers used above are listed in Table S11. The MdNAC1-BD fusion protein, pGBKT7 vector (negative control), and positive control were individually transformed into the yeast strain AH109. After being cultured on Trp-free media, the yeast cells were transferred to the SD-Trp-His condition and finally added to the X-α-Gal solution. The growth and staining results of yeast cells can be used as a reference for self-activated activity.

#### Transient overexpression and VIGS

The *MdEXP-A1* CDS was amplified from ‘Golden Delicious’ fruits and inserted into the pCAMBIA2300 vector with a GFP label using double restriction enzyme site (*Kpn*I and *Bamh*I) to generate the recombinant constructs. The primers used are shown in Table S11. The specific fragment (400 bp) of the *MdEXP-A1* CDS was subsequently cloned and inserted into the pTRV2 carrier for gene silencing. The control and fusion plasmids were subsequently individually transferred into the GV3101 *Agrobacterium* strain. The methods used for the transformation of apple fruits were previously reported (Wang et al. 2023b). The relative expression of the samples subjected to transient transformations was determined via RT‒qPCR.

### Determination of cell wall components

The total cell wall material was extracted according to Su et al. (2022). In brief, frozen samples (5.0 g) were subjected to crude CWM extraction via dissolution in acetone and dimethyl sulfoxide. Then, distinct types of pectin polysaccharides were extracted via sodium acetate, (1, 2- cyclohexylenedinitrilo)-tetra acetic acid, and Na_2_CO_3_ (0.05 M) and determined via the carbazole ethanol method. Hemicellulose was subsequently extracted with KOH (4 M), which was measured via anthrone-sulfuric acid method. The resulting cell wall residue was dominated by cellulose.

### Genetic transformation of apple calli and Micro-Tom

Apple calli was isolated from the ‘Orin’ embryo, which was subcultured every two weeks. The CDSs of *MdNAC1* and *MdEXP-A1* were connected to the pCAMBIA2300 vector for stable overexpression. Then, the *MdNAC1* and *MdEXP-A1* constructs were separately transferred into EHA105 *Agrobacterium*, and apple calli transformation of *MdNAC1* was conducted on the basis of Zhang et al. (2022a). The tomato transformation of *MdEXP-A1* was performed using the same method described previously (Fillatti et al. 1987). The positive seedlings were confirmed via PCR with detection primers (Table S11).

### Yeast one hybrid assay

The Y1H assay was accomplished as previously described (Zhang et al. 2023b). The CDS of *MdNAC1* was cloned and inserted into the pGADT7 effector carrier. The *MdEXP-A1* promoter fragment (P-2) was linked to the pAbiA vector (reporter). The primers used for Y1H are listed in Table S11. Y1H yeast cells harboring the *MdEXP-A1-*P-2-pAbiA plasmid were cultured on SD-Ura medium, supplemented with aureobasidin A (AbA) at different concentrations to inhibit self-activating activity. The pGADT7-*MdNAC1* construct was subsequently co-transformed into a Y1H cells containing *MdEXP-A1-*P-2-pAbiA. Next, the transformation products were transferred to screening medium lacking Leu supplemented with the corresponding AbA. The interaction results were evaluated by observing the growth of the strain carrying the pGADT7 vector as a control.

### Electrophoretic mobility shift assay

The EMSA experiment was carried out as previously outlined (Wang et al. 2023b). The *MdNAC1* CDS was linked into the pGEX-6p-1 vector and transformed into BL21 (DE3) *Escherichia coli* for GST-tag protein expression. Specific binding, competition, and mutation probes were obtained on the basis of the sequence of the *MdEXP-A1* deletion fragment. The above probes were synthesized and processed at Sangon Biotech. EMSA was performed with a Chemiluminescent EMSA Kit (GS009, Beyotime). The primers used are shown in Table S11.

### Transient dual-luciferase assay

For the dual-luciferase experiment, the *MdNAC1* CDS was merged into the pGreenII 62-SK vector driven by CaMV35S (the effector). The full-length promoter of *MdEXP-A1* (2179 bp) was integrated into pGreenII 0800-LUC (the reporter). All the constructs and empty vectors were introduced into the GV3101 strain harboring the pSoup vector. Then, they were transiently infected with one-month-old tobacco leaves. After three days of transformation, the LUC signal and activity were measured as previously described (Fu et al. 2021).

### GUS staining analysis

To investigate whether the 1166 bp variation in the *MdEXP-A1* promoter affects its transcriptional activity, truncated promoter fragments (Pro1809, Pro1049, and Pro643) were amplified from the DNA of ‘Golden Delicious’ through PCR. As previously described, a pCAMBIA1301 reporter vector without 35S was constructed with different segments of the above *MdEXP-A1* promoter via restriction enzyme sites (*Hind*III and *EcoR*V). Three recombinant plasmids and the pCAMBIA1301 empty vector were transformed into to *Agrobacterium* GV3101 which was subsequently injected into tobacco leaves (one-month-old). Three days after injection, the leaves were clipped and stained with a GUS stain kit (SL7160, Coolaber) for 12h. Each experiment was repeated more than three times. All primers used are listed in Table S11.

### Statistical analysis

The SPSS software (IBM) was used for statistical analysis. The data were analyzed according to a one-way ANOVA followed by Student’s *t* test analysis. Significant differences are indicated with asterisks (*P < 0.05, **P < 0.01).

## Supplementary Information

Additional file 1: Supplementary Figure S1 QTL analysis for flesh firmness at harvest. Manhattan plot for flesh firmness. Supplementary Figure S2. Heatmap of differentially expressed genes in the QTL region for apple flesh firmness at harvest. Supplementary Figure S3 Heatmap of candidate genes involved in cell wall metabolism at diverse developmental stages. Supplementary Figure S4 The alignment of the coding domain sequence (CDS) of *MdEXP-A1 *between the ‘Ruiyang’ cultivar and the ‘Golden Delicious’ genome. Supplementary Figure S5 Comparative genomic analysis of the *MdEXP-A1* upstream promoter in both extra-hard and extra-soft bulks via IGV software and next-generation sequencing. Supplementary Figure S6 Analysis of the alignment of the promoters of *MdEXP-A1* between the ‘Ruiyang’ and ‘Golden Delicious’ reference genome. Supplementary Figure S7 The apple genome annotation revealed the presence of transposable elements with 1145 bp upstream of *MdEXP-A1*. Supplementary Figure S8 The BLAST results of the 1166 bp transposon sequence suggest the presence of numerous similar fragment sequences across different chromosomes of the apple genome. Supplementary Figure S9 Amino acid sequence alignments of homologous MdEXP-A1 proteins in diverse species. Supplementary Figure S10 Different stages and identification of the *MdEXP-A1* transgenic tomato plants. Supplementary Figure S11 Analysis of *cis*-acting elements in the TE-1166 locus of the *MdEXP-A1 *promoter sequence. Supplementary Figure S12 Analysis of *cis*-acting elements in the full-length *MdEXP-A1* promoter sequence. Supplementary Figure S13 Identification of the expression of* MdNAC1 *in wild-type (WT) and stable transgenic calli via RT‒PCR. Supplementary Figure S14 RNA-seq analysis of differentially expressed genes (DEGs) between WT and OE-*MdNAC1* transgenic calli.

Additional file 2: Supplementary Table S1. The firmness data of F_1_ hybrids from the cross of ‘Ruiyang’ and ‘Scilate’ in 2021. Supplementary Table S2. The firmness data of F_1_ hybrids from the cross of ‘Ruiyang’ and ‘Scilate’ in 2022. Supplementary Table S3. Genetic parameters of flesh firmness in the progeny of ‘Ruiyang’ and ‘Scilate’. Supplementary Table S4. *MdACO1* genotype in 40 F_1_ super-hard and super-soft progeny from a cross of ‘Ruiyang’ and ‘Scilate’. Supplementary Table S5. *MdACS1* genotype in F_1_ super-hard and super-soft progeny from a cross of ‘Ruiyang’ and ‘Scilate’. Supplementary Table S6. List of DEGs in the QTL region for flesh firmness at harvest. Supplementary Table S7. List of candidate genes involved in cell wall metabolism and TFs from the QTL region for flesh firmness. Supplementary Table S8. Developmental expression of candidate genes related to cell wall metabolism. Supplementary Table S9. List of the TE-1166 genotype in extra-hard and extra-soft F_1_ hybrids from ‘Fuji’ and ‘Cripps Pink’. Supplementary Table S10. List of the TE-1166 genotype in super-hard and super-soft F_1_ hybrids from ‘Ruiyang’ and ‘Scilate’. Supplementary Table S11. List of primers used in this study.

## Data Availability

Transcriptome date during development was derived from Liu et al. (2021) and are available in the NCBI database: the accession number is PRJNA728501. Sequence data used in this article can be found in the GenBank data libraries under accession numbers: *MdEXP-A1* (MD16G1070600), *MdNAC1* (MD13G1069200), *MdACS1* (MD15G1302200), *MdACO1* (MD10G1328100), *MdPG1* (MD10G1179100).

## Abbreviations

ABA: Abscisic acid
AbA: Aureobasidin A
BSA-seq: Bulked segregant analysis sequencing
CDS: Coding sequence
DEGs: Differentially expressed genes
EMSA: Electrophoretic mobility shift assay
ETH: Ethylene
FPKM: Fragments per kilobase per million
InDels: Insertion-deletion
LUC: Luminescence
MeJA: Jasmonic acid methylester
NAA: Naphthylacetic acid
RNA-seq: RNA-sequencing
RT-qPCR: Reverse transcription-quantitative polymerase chain reaction
SNP: Single nucleotide polymorphism
TEs: Transposable elements
TFs: Transcription factors
WT: Wild-type
Y1H: Yeast one-hybrid
