# Supplementary material for: Allelic variation in an expansin, MdEXP-A1, contributes to flesh firmness at harvest in apples
Source: Mol Hortic. 2025 Jan 20;5:3. doi: 10.1186/s43897-024-00121-3 (PMC11744834; doi:10.1186/s43897-024-00121-3)
Supplement: Supplementary file 1 — Additional file 1: Supplementary Figure S1 QTL analysis for flesh firmness at harvest. Manhattan plot for flesh firmness. Supplementary Figure S2. Heatmap of differentially expressed genes in the QTL region for apple flesh firmness at harvest. Supplementary Figure S3 Heatmap of candidate genes involved in cell wall metabolism at diverse developmental stages. Supplementary Figure S4 The alignment of the coding domain sequence (CDS) of MdEXP-A1 between the ‘Ruiyang’ cultivar and the ‘Golden Delicious’ genome. Supplementary Figure S5 Comparative genomic analysis of the MdEXP-A1 upstream promoter in both extra-hard and extra-soft bulks via IGV software and next-generation sequencing. Supplementary Figure S6 Analysis of the alignment of the promoters of MdEXP-A1 between the ‘Ruiyang’ and ‘Golden Delicious’ reference genome. Supplementary Figure S7 The apple genome annotation revealed the presence of transposable elements with 1145 bp upstream of MdEXP-A1. Supplementary Figure S8 The BLAST results of the 1166 bp transposon sequence suggest the presence of numerous similar fragment sequences across different chromosomes of the apple genome. Supplementary Figure S9 Amino acid sequence alignments of homologous MdEXP-A1 proteins in diverse species. Supplementary Figure S10 Different stages and identification of the MdEXP-A1 transgenic tomato plants. Supplementary Figure S11 Analysis of cis-acting elements in the TE-1166 locus of the MdEXP-A1 promoter sequence. Supplementary Figure S12 Analysis of cis-acting elements in the full-length MdEXP-A1 promoter sequence. Supplementary Figure S13 Identification of the expression of MdNAC1 in wild-type (WT) and stable transgenic calli via RT‒PCR. Supplementary Figure S14 RNA-seq analysis of differentially expressed genes (DEGs) between WT and OE-MdNAC1 transgenic calli. [file 43897_2024_121_MOESM1_ESM.pdf]

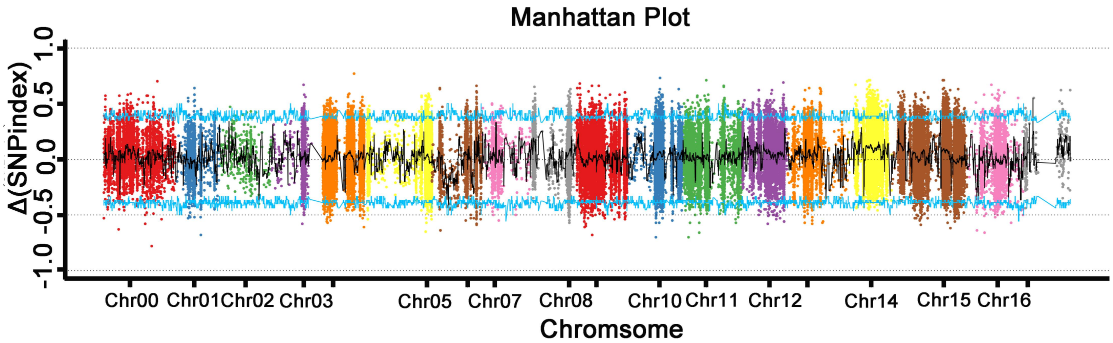

Supplementary Figure S1 QTLs analysis for flesh firmness at harvest from a cross of ‘Ruiyang’ and ‘Scilate’. Manhattan plot for flesh firmness.

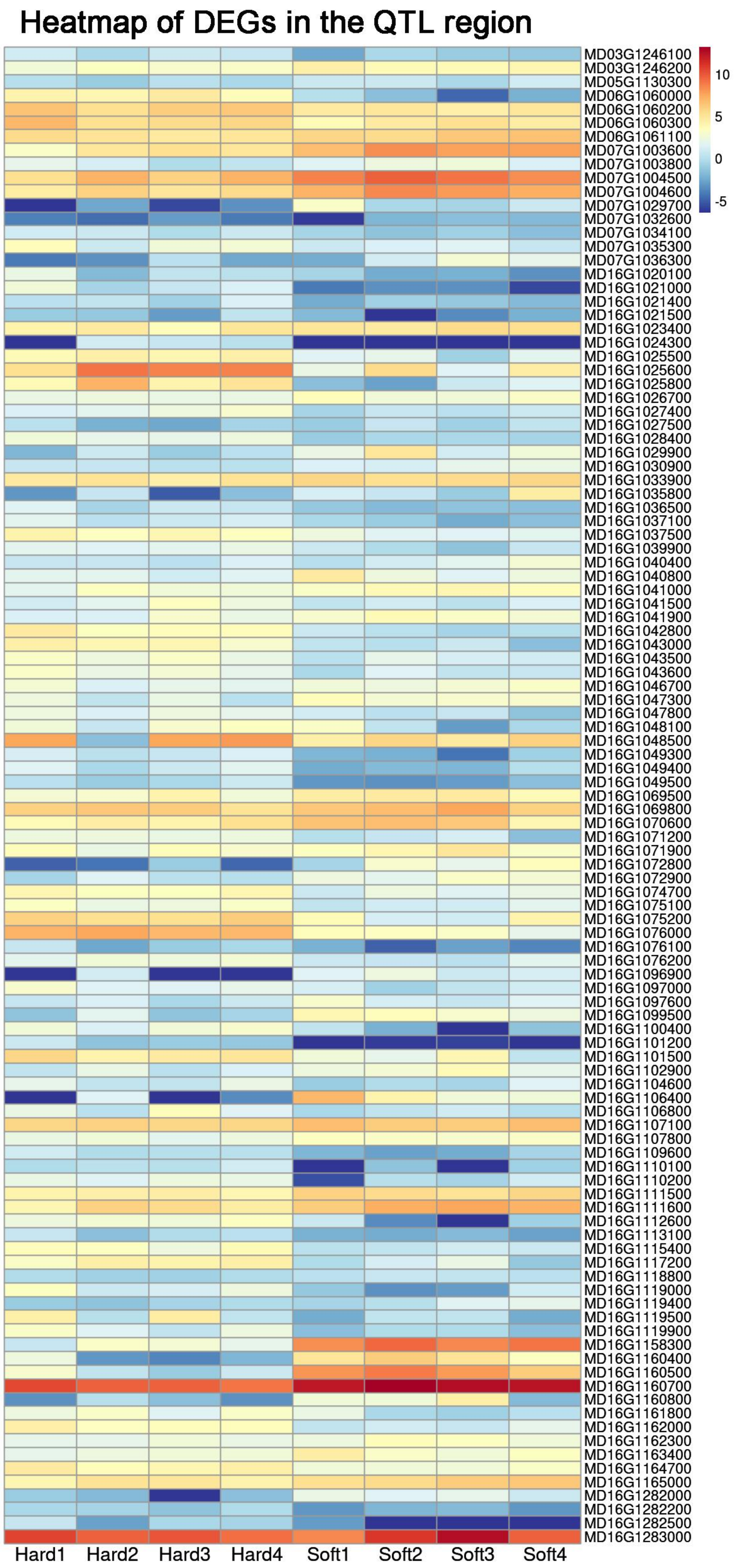

Supplementary Figure S2 Heatmap of differentially expressed genes in the QTL region for apple flesh firmness at harvest.

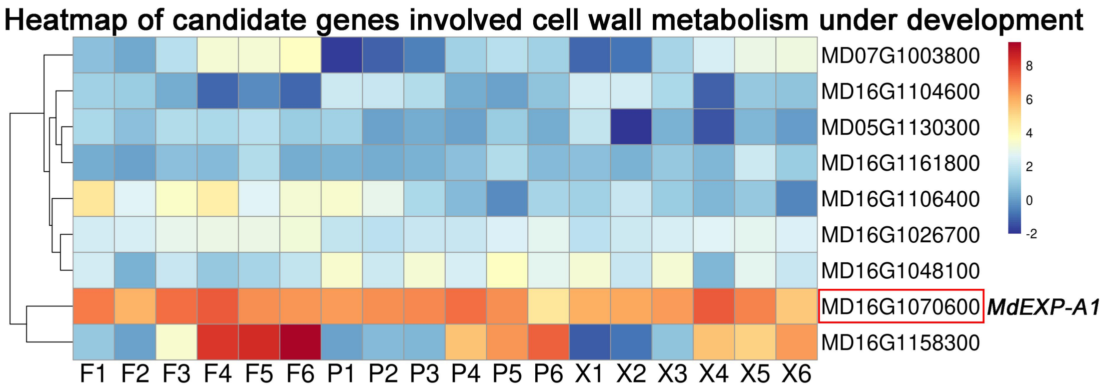

Supplementary Figure S3 Heatmap of candidate genes involved in cell wall metabolism at diverse developmental stages.

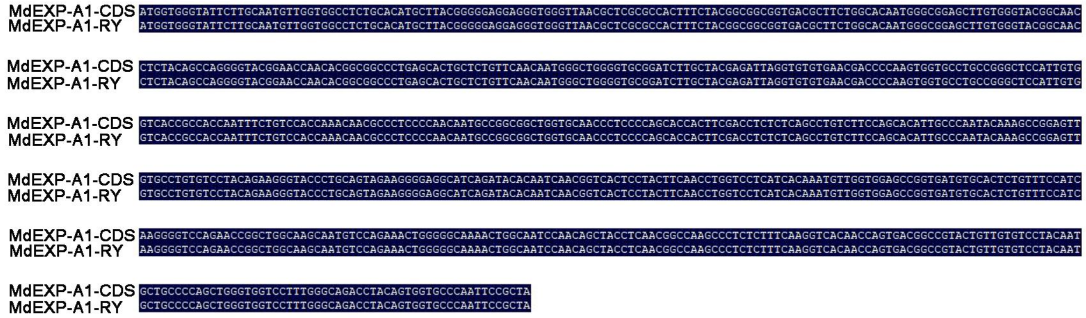

Supplementary Figure S4 The alignment of the coding domain sequence (CDS) of *MdEXP-A1* between the 'Ruiyang' cultivar and the 'Golden Delicious' genome.

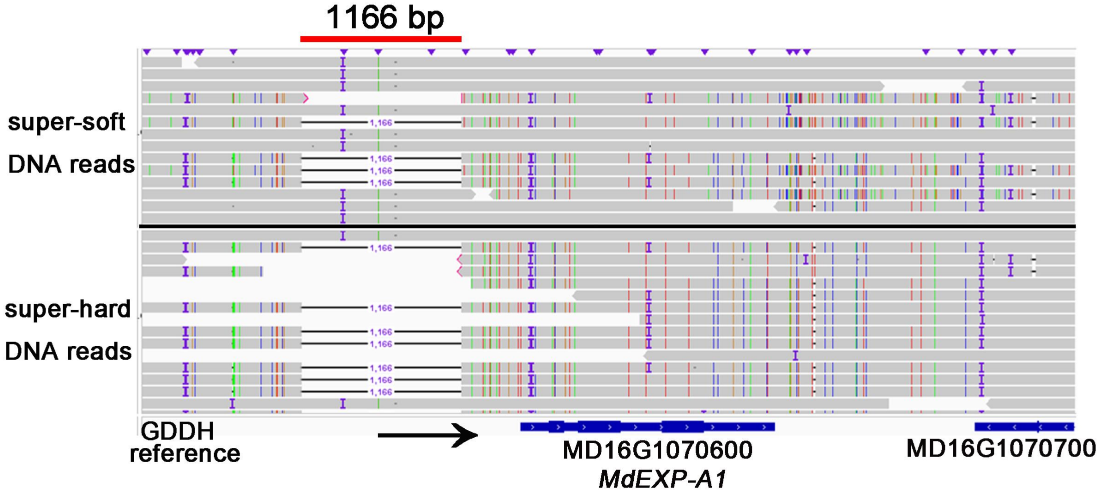

Supplementary Figure S5 Comparative genomic analysis of *MdEXP-A1* upstream promoter in both extra-hard and extra-soft pools via IGV software and next-generation sequencing (unpublished data in our lab)

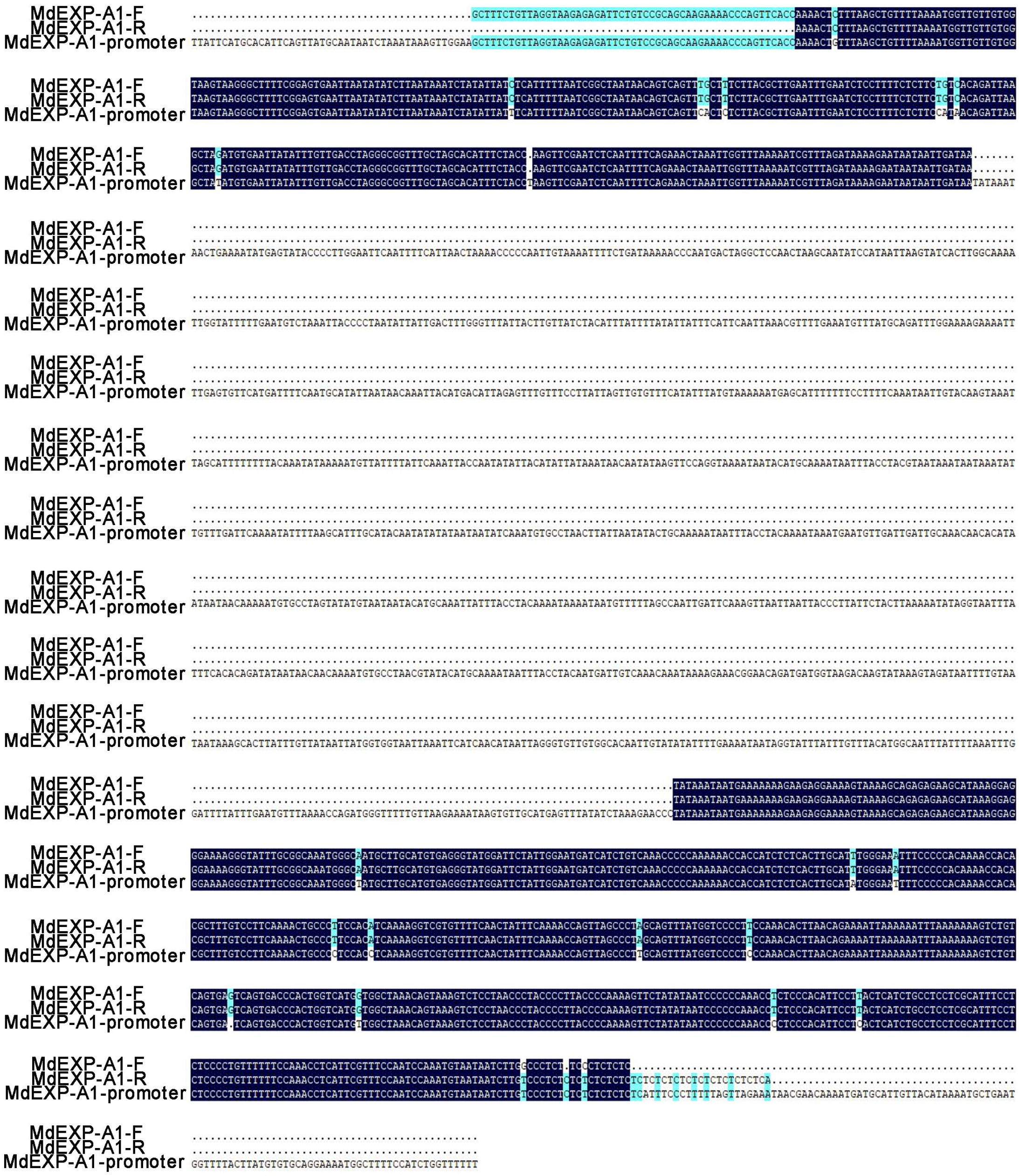

Supplementary Figure S6 Analysis of the alignment of the promoters of *MdEXP-A1* between the ‘Ruiyang’ and ‘Golden Delicious’ reference genome.

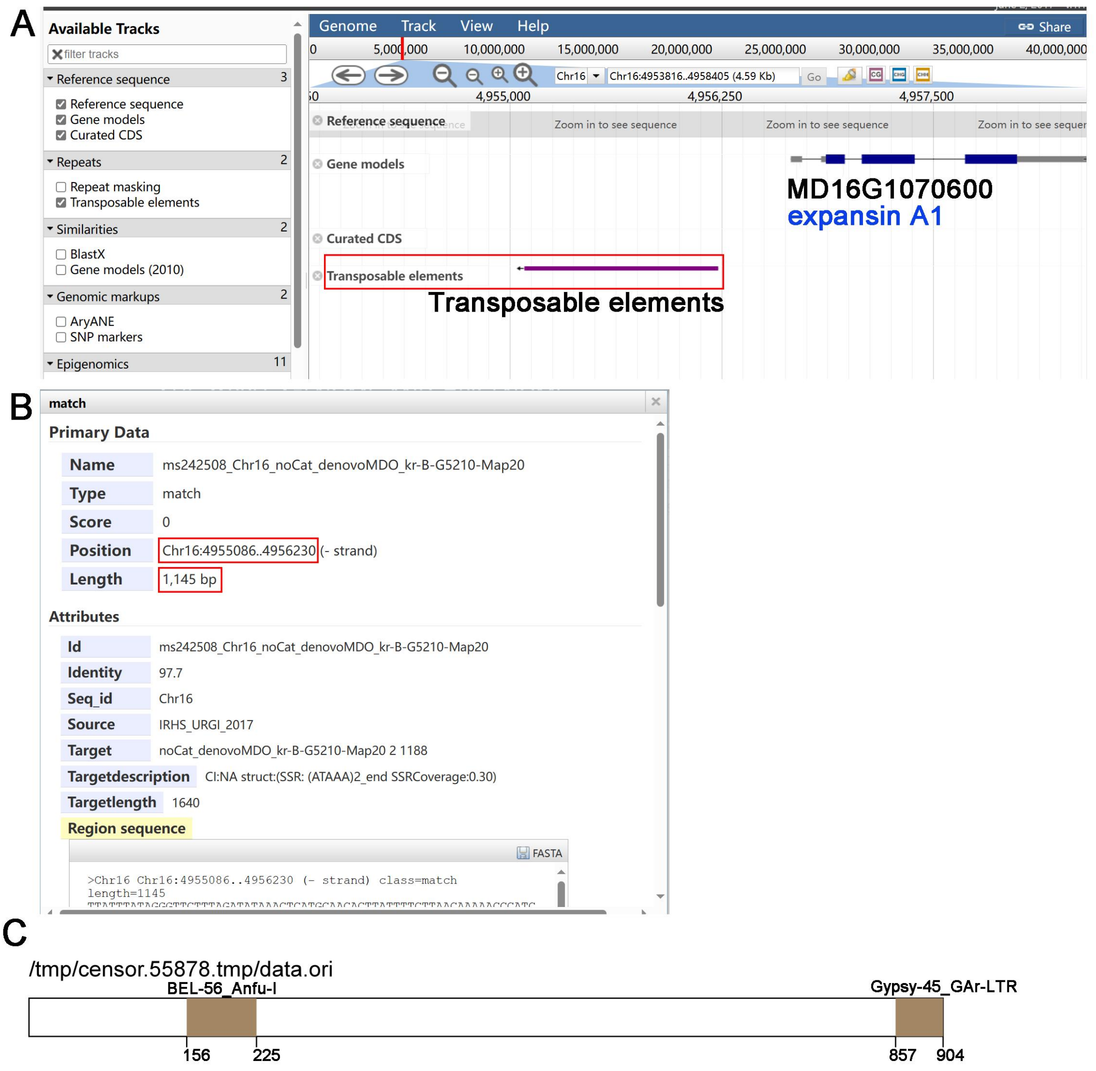

Supplementary Figure S7 The apple genome annotation revealed the presence of a transposable element with 1145 bp upstream of *MdEXP-A1*. **A** The apple genome annotation of MdEXP-A1 region. **B** The genome annotation of the 1145bp transposable element. **C** TE-1166 contains one LTR/BEL and one LTR/Gypsy family transposon.

Malus domestica genome assembly, chromosome: 16	Malus domestica	2019	11696	100%	0.0	97.80%	40503753	OU744557.1
Malus sylvestris genome assembly, chromosome: 16	Malus sylvestris	1984	12374	100%	0.0	97.30%	40115383	OU696518.1
Malus domestica genome assembly, chromosome: 9	Malus domestica	1960	7763	99%	0.0	97.11%	35912611	OU744961.1
Malus sylvestris genome assembly, chromosome: 9	Malus sylvestris	1958	6245	99%	0.0	96.95%	35179981	OU696511.1
Malus x robusta genome assembly, chromosome: 14	Malus x robusta	1956	4511	99%	0.0	96.95%	31419793	OY720338.1
Malus domestica genome assembly, chromosome: 6	Malus domestica	1956	4366	99%	0.0	96.95%	31763870	OU744996.1
Malus domestica genome assembly, chromosome: 6	Malus domestica	1956	6288	99%	0.0	96.95%	35607933	OU744958.1
Malus sylvestris genome assembly, chromosome: 6	Malus sylvestris	1956	4432	99%	0.0	96.95%	34960253	OU696508.1
Malus domestica genome assembly, chromosome: 9	Malus domestica	1956	4053	99%	0.0	97.03%	37073583	OU696685.1
Malus domestica genome assembly, chromosome: 4	Malus domestica	1895	4238	100%	0.0	96.17%	31483556	OU744994.1
Malus x robusta genome assembly, chromosome: 17	Malus x robusta	1884	6525	100%	0.0	96.00%	25717622	OY720341.1
Malus x robusta genome assembly, chromosome: 13	Malus x robusta	1882	7732	100%	0.0	96.76%	31897208	OY720337.1
Malus domestica genome assembly, chromosome: 12	Malus domestica	1882	6903	100%	0.0	96.76%	32233215	OU744964.1
Malus sylvestris genome assembly, chromosome: 4	Malus sylvestris	1869	4410	100%	0.0	95.74%	31139927	OU696506.1
Malus domestica genome assembly, chromosome: 12	Malus domestica	1866	7897	100%	0.0	96.50%	32853497	OU744553.1
Malus sylvestris genome assembly, chromosome: 12	Malus sylvestris	1866	6870	100%	0.0	96.50%	32552200	OU696514.1
Malus domestica genome assembly, chromosome: 4	Malus domestica	1772	4706	100%	0.0	96.59%	31281846	OU696680.1
Malus x robusta genome assembly, chromosome: 3	Malus x robusta	1533	6342	99%	0.0	90.87%	40965740	OY720327.1
Malus sylvestris genome assembly, chromosome: 13	Malus sylvestris	1435	6383	99%	0.0	94.06%	44069681	OU696515.1
Malus domestica genome assembly, chromosome: 3	Malus domestica	1424	8109	100%	0.0	93.61%	38144444	OU696679.1
Malus domestica genome assembly, chromosome: 6	Malus domestica	1421	4464	99%	0.0	93.85%	35541377	OU744547.1
Malus domestica genome assembly, chromosome: 6	Malus domestica	1421	4361	99%	0.0	93.85%	35846082	OU696682.1
Malus domestica genome assembly, chromosome: 2	Malus domestica	1417	4236	99%	0.0	93.75%	38036873	OU744992.1
Malus domestica genome assembly, chromosome: 2	Malus domestica	1417	4254	99%	0.0	93.75%	37910991	OU696678.1
Malus domestica genome assembly, chromosome: 1	Malus domestica	1413	3140	99%	0.0	93.49%	30155919	OU696677.1
Malus domestica genome assembly, chromosome: 16	Malus domestica	1406	11870	100%	0.0	93.54%	41419100	OU745006.1
Malus domestica genome assembly, chromosome: 16	Malus domestica	1406	12060	100%	0.0	93.54%	40049923	OU696692.1
Malus domestica genome assembly, chromosome: 3	Malus domestica	1402	4184	99%	0.0	93.45%	37047584	OU744955.1
Malus sylvestris genome assembly, chromosome: 3	Malus sylvestris	1402	3814	99%	0.0	93.45%	36902754	OU696505.1
Malus x robusta genome assembly, chromosome: 8	Malus x robusta	1397	4187	99%	0.0	93.34%	35755900	OY720332.1
Malus domestica genome assembly, chromosome: 9	Malus domestica	1397	4247	99%	0.0	93.34%	35819836	OU744999.1
Malus domestica genome assembly, chromosome: 9	Malus domestica	1397	4190	99%	0.0	93.34%	36124330	OU744550.1
Pyrus communis genome assembly, chromosome: 13	Pyrus communis	1395	4851	99%	0.0	88.29%	25886306	OY757080.1
Malus x robusta genome assembly, chromosome: 10	Malus x robusta	1384	10714	100%	0.0	93.06%	34111749	OY720334.1

Supplementary Figure S8 The BLAST results of the 1166 bp transposon sequence suggest the presence of numerous similar fragment sequence across different chromosomes of the apple genome.

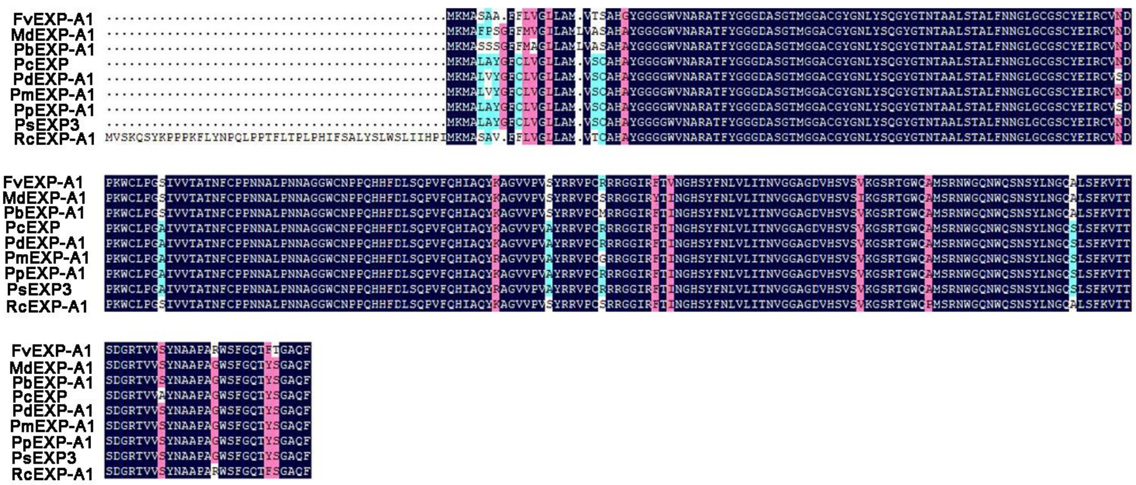

Supplementary Figure S9 Amino acid sequence alignment of homologous MdEXP-A1 proteins in diverse species. The protein sequence number is downloaded from NCBI (https://www.ncbi.nlm.nih.gov/)

FvEXP-A1： XP_004297292.1； MdEXP-A1： XP_008361782.1; PbEXP-A1： XP_009360325.1; PcEXP ： AAL40354.1; PdEXP-A1: XP_034210113.1;PmEXP-A1:XP_008221599.1 ; PpEXP-A1:XP_007226005.1; PsEXP3:AEQ28765.1; RcEXP-A1：XP_024195326.1.

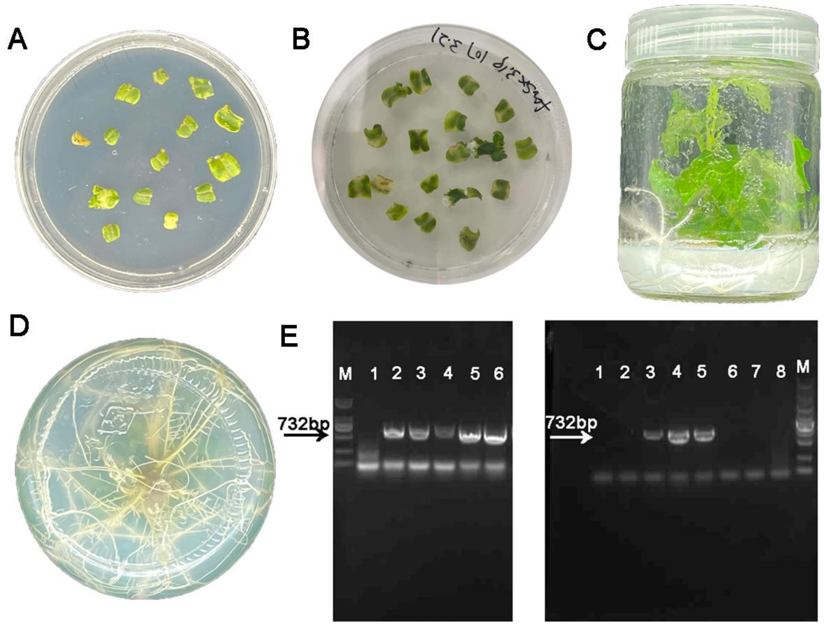

Supplementary Figure S10 Different stages and identification of the *MdEXP-A1* transgenic tomato plants. **A** Pre-culture **B** Co-culture **C** Rooting culture **D** Root phenotype **E** Identification of *MdEXP-A1* in wild-type (WT) and stable transgenic tomatoes.

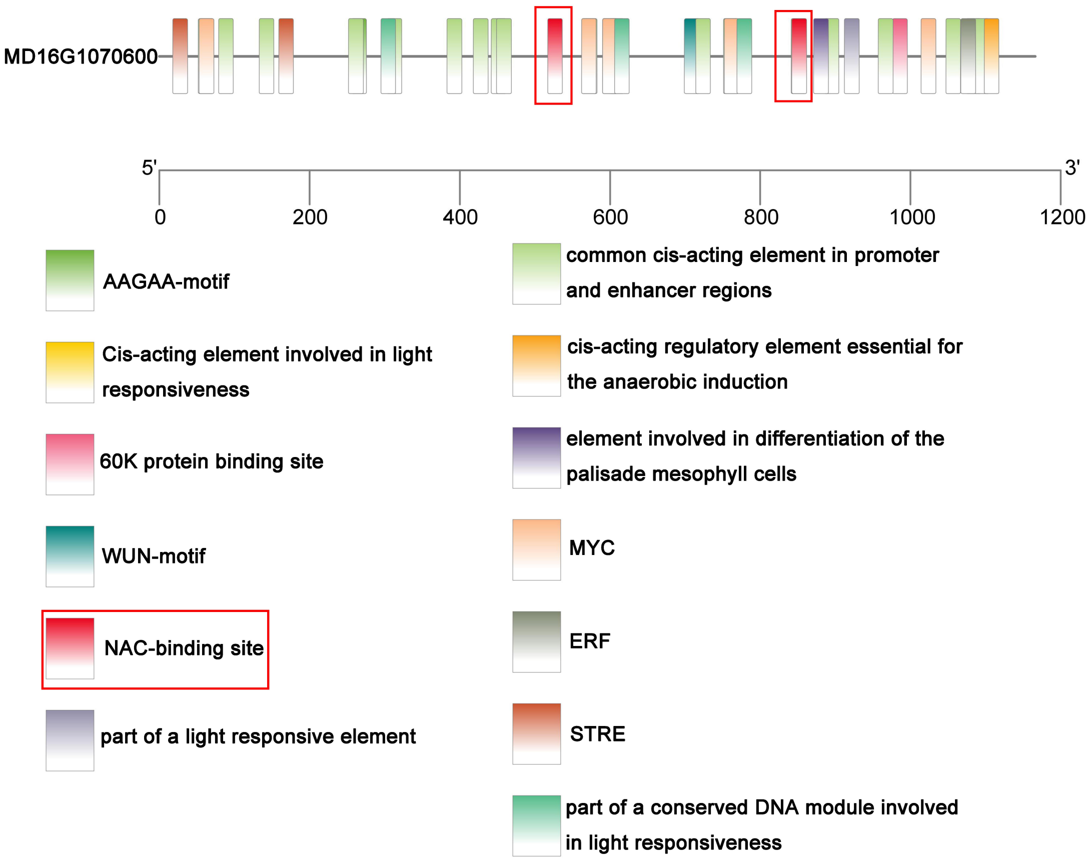

Supplementary Figure S11 Analysis of cis-acting elements in the TE-1166 locus of the *MdEXP-A1* promoter sequence. [ACGTA] is a cis acting element specially combined by NAC proteins.

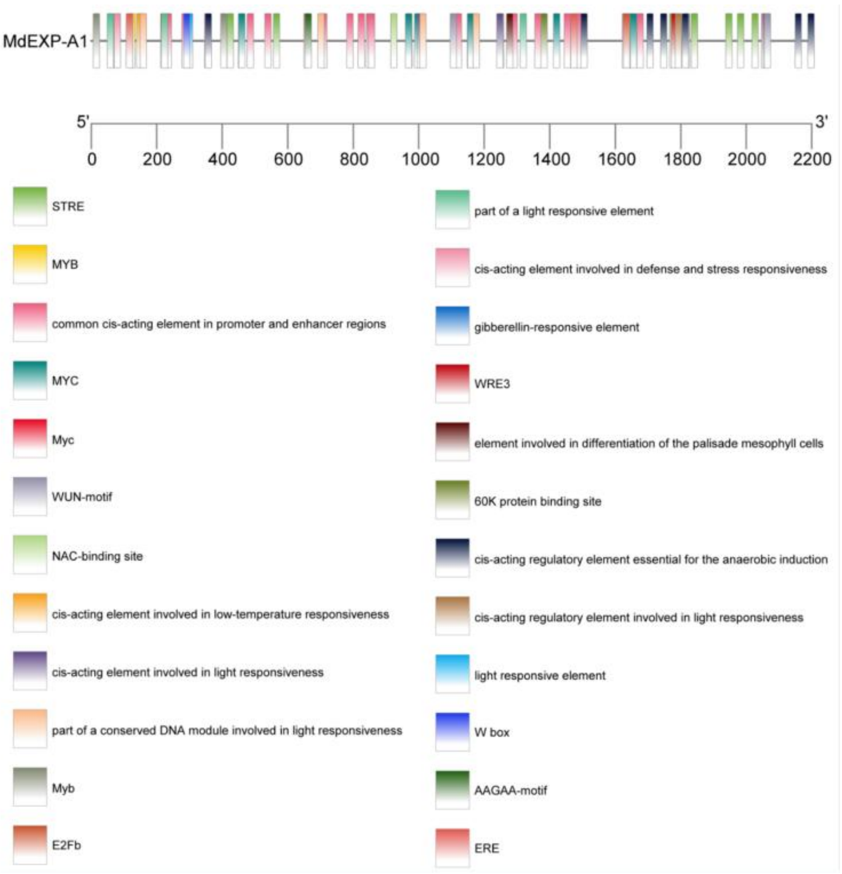

Supplementary Figure S12 Analysis of cis-acting elements in the full-length *MdEXP-A1* promoter sequence (2207 bp).

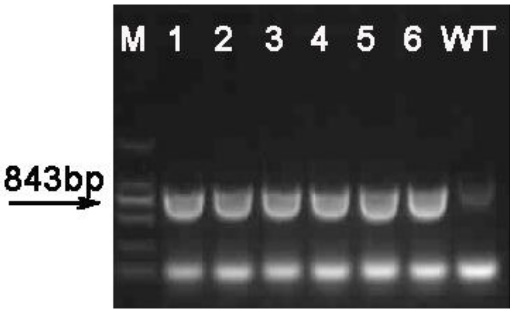

Supplementary Figure S13 Identification of the expression of *MdNAC1* in wild-type (WT) and stable transgenic calli by RT–PCR.

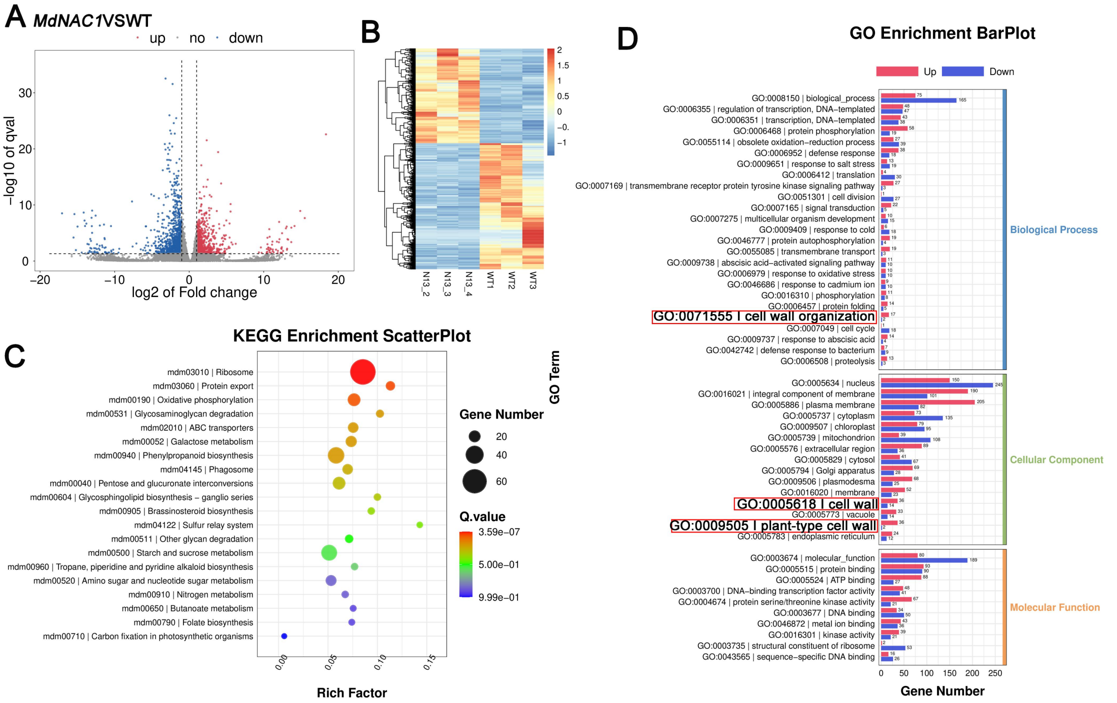

Supplementary Figure S14 RNA-seq analysis of differentially expressed genes (DEGs) between WT and OE-*MdNAC1* transgenic calli. **A** Volcano plot of DEGs. **B** Hierarchical cluster analysis of DEGs in WT and OE-*MdNAC1* transgenic calli. **C** KEGG pathway enrichment of DEGs. **D** GO term classifications of DEGs.
